# Distinct genetic architecture in the tails of complex traits

**DOI:** 10.1038/s41586-026-10516-5

**Published:** 2026-05-27

**Authors:** T. Souaiaia, H. M. Wu, A. P. S. Ori, S. W. Choi, C. J. Hoggart, P. F. O’Reilly

**Affiliations:** 1 Department of Cellular Biology, SUNY Downstate Health Sciences University, Brooklyn, NY USA; 2 Department of Genetics and Genomic Sciences, Icahn School of Medicine, Mount Sinai, New York, NY USA; 3 Department of Psychiatry, Amsterdam UMC, University of Amsterdam, Amsterdam, the Netherlands

**Keywords:** Genetic variation, Genetic association study, Evolutionary genetics, Statistical methods, Rare variants

## Abstract

Complex traits are highly polygenic, with heritability explained by many hundreds of common variants of small effect together with rare variants of large effect^1^. Yet how this genetic architecture varies along the trait continuum has been underexplored, as has the role of natural selection in shaping this variation. Here we developed an approach based on polygenic risk scores that reveals widespread departures from common-variant architecture in one or both of the tails of 74 quantitative traits. These observations were replicated across ancestries, cohorts and repeated measures and using an alternative family-based approach^2^. Incorporating rare variants identified from sequence data resulted in marked reductions in these deviations, suggesting that rare alleles of large effect are key drivers of trait-tail architecture. Forward simulations showed that stabilizing selection could generate the observed patterns, whereas modelling reproductive success provided empirical support for the role of selection. These findings show that although complex traits are polygenic in the population at large, they have a distinct and less polygenic architecture in their tails due to selection. This has implications for rare-variant discovery and complex trait and disease prediction.

## Abstract (web summary)

Genome-wide analyses in multiancestry and European cohorts show that in complex traits, rare alleles have disproportionately large effects at the tails of the phenotypic spectrum compared with common alleles.

## Main

The number of genetic variants identified by genome-wide association and sequencing studies is now so large that a complete picture of the genetic architecture of many complex traits is starting to emerge^1,3^. Illustrating all known variants for a complex trait or disease on a spectrum of allele frequency versus effect size produces a characteristic ‘trumpet’ shape^4,5^, in which rarer alleles have increasingly larger effects owing to stabilizing selection^6^. These effects are sometimes so large relative to those of common alleles that they cause extreme trait values on most genetic backgrounds. For example, mutations in *ACAN* usually result in short stature, those in *LEP* cause leptin deficiency, and those in *HNF1A* produce large deviations in glucose homeostasis^7–9^. Moreover, Fiziev et al. found that their PrimateAI-derived rare-variant polygenic risk scores (PRSs) were particularly predictive for individuals with phenotypic extremes^10^ and rare alleles with marked effects on complex disease have also been identified^11,12^.

**Fig. 1:**
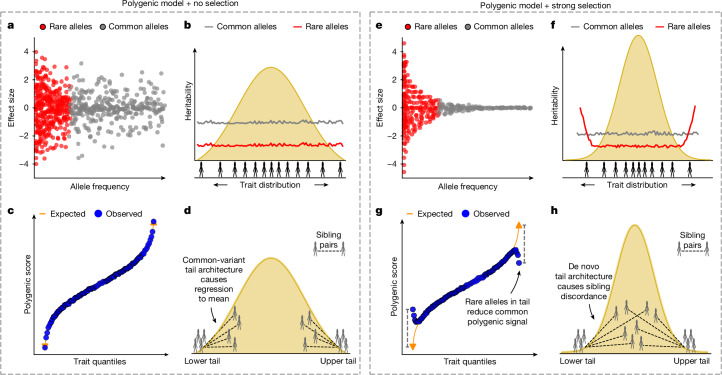
Two polygenic models of complex trait genetic architecture. **a**–**d**, In the polygenic model with no selection: the minor allele frequency (MAF) and effect size of variants affecting the trait are uncorrelated (**a**); common and rare variants are uniformly distributed across the trait continuum and common variants explain more heritability (**b**); the trait and common-variant polygenic score have a linear relationship (**c**); and siblings of individuals in the tails of the trait distribution also show low/high trait values, with regression to the mean to an extent dependent on trait heritability (**d**)^2^. In this model, common-variant architecture predominates the entire trait distribution. **e**–**h**, In the polygenic model with strong selection (here, stabilizing selection): there is a strong negative exponential relationship between MAF and effect size (**e**); common variants explain more heritability overall, but rare variants contribute more in the tails (**f**); the common-variant polygenic score regresses to the mean in the trait tails owing to large-effect rare alleles not included in the polygenic score (**g**); and under selection so strong that tails are characterized by de novo architecture, siblings of individuals in the trait tails have trait values drawn from the background distribution (**h**). In this model, common-variant architecture predominates in the body of the trait distribution, whereas rare-variant architecture prevails in the tails. These models illustrate two extremes of selection that are unrealistic in practice, but most complex traits should have genetic architecture that varies across the trait continuum in a manner between these two extremes.

Given the importance of natural selection in shaping the joint distribution of allele frequency and effect size (Fig. 1a,e), the relative contribution of common and rare variants may vary across the trait continuum according to historical selective pressures. Consequently, we would expect selection-dependent variation in the distribution of heritability (Fig. 1b,f), of PRSs (Fig. 1c,g) and of trait similarity within families (Fig. 1d,h) across the trait continuum. In practice, selective pressures vary over time, so a trait broadly subject to stabilizing selection may have also undergone intermittent periods of directional selection towards new optimal values as environments changed^13^. Moreover, complex traits are highly pleiotropic, meaning that selection on most variants is integrated over multiple traits^14^. Nonetheless, a range of realistic forms of selection on human complex traits may be expected to produce broadly similar patterns, albeit with variation in magnitude and symmetry across the trait distribution.

On the basis of the existence of rare alleles that can alone generate extreme trait values, as well as growing evidence that stabilizing selection is a pervasive driver of complex trait architecture^6,15,16^, we proposed that stabilizing selection on some traits may have been sufficiently strong to have produced marked enrichments of rare alleles of large effect in their tails. The magnitude of such enrichments would depend on the number, effect sizes and frequencies of these alleles across the genome, whereas their symmetry would depend on the relative contributions of stabilizing and directional selection in recent history. Motivated by the expected differences in genetic architecture across traits subject to different forms and strengths of selection (Fig. 1), we used two complementary approaches to test for tail-specific departures from common-variant architecture: first, we introduced the PRS-on-phenotype outlier test (POPout), which evaluates whether PRS values in the lower and upper tails depart from a linear relationship fitted across the trait–PRS distribution; and, second, we extended a previously developed family-based framework^2^ to derive STANDout, a test based on differences between individuals in the tails and their siblings, to detect rare-variant tail architecture. We applied these methods to 74 quantitative traits from the UK Biobank (UKB), tested generalizability by replication across repeated measures, multiple ancestries and the All of Us Research Program, and then assessed direct evidence for the role of rare variants using whole-exome sequencing (WES) and whole-genome sequencing (WGS) data. Finally, we tested whether selection could generate the observed signals using forward simulations^17^ and empirical inference of selection from lifetime reproductive success^16^. Our findings reveal distinct genetic architecture in the tails of complex traits, highlight the role of selection in the causes of phenotypic extremes, and suggest strategies for increasing the discovery of rare variants and improving the accuracy of complex trait and disease prediction.

## Widespread POPout effects

We analysed quantitative traits in the UKB^18^, covering a broad range of blood biomarkers and physical, cognitive and behavioural measures. After quality control (QC) to ensure, for example, adequate heritability, polygenicity and normality of the traits, 74 traits were selected for analysis (Methods). To limit the contribution of environmental factors, we residualized traits for relevant covariates and removed individuals who were diagnosed with cancer, were on statins or insulin medication, or were pregnant. The primary analysis included up to 369,132 individuals of European ancestry. For each trait, we performed genome-wide association studies (GWAS) in half the sample and computed common-variant PRSs in the other half, and then tested for tail-specific deviations in PRSs using POPout (Methods). This design separates discovery from scoring while retaining sufficiently large tail sample sizes.

POPout inverts standard PRS analyses^19^: instead of evaluating how phenotype varies across PRS quantiles^20,21^, POPout tests how PRS varies over phenotype quantiles, providing insight into variation in genetic architecture across the trait distribution. Under a null model in which common-variant architecture predominates the entire distribution, PRS should increase linearly with phenotype. Under a model in which high-effect rare alleles concentrate in the tails, individuals with extreme phenotypes will, on average, carry fewer common risk alleles than expected for their phenotype, producing regression of common PRS towards the mean in the tails. We quantify this deviation by testing whether PRS residuals in the lower and upper tails differ from expectations on the basis of a regression fit over the entire trait distribution, after verifying calibration in the body of the distribution (Methods). In primary analyses, the POPout test was applied to the lower and upper 1% tails (see description of sensitivity analyses), which balances extremeness with statistical power while reflecting prevalence rates of complex diseases.

**Fig. 2:**
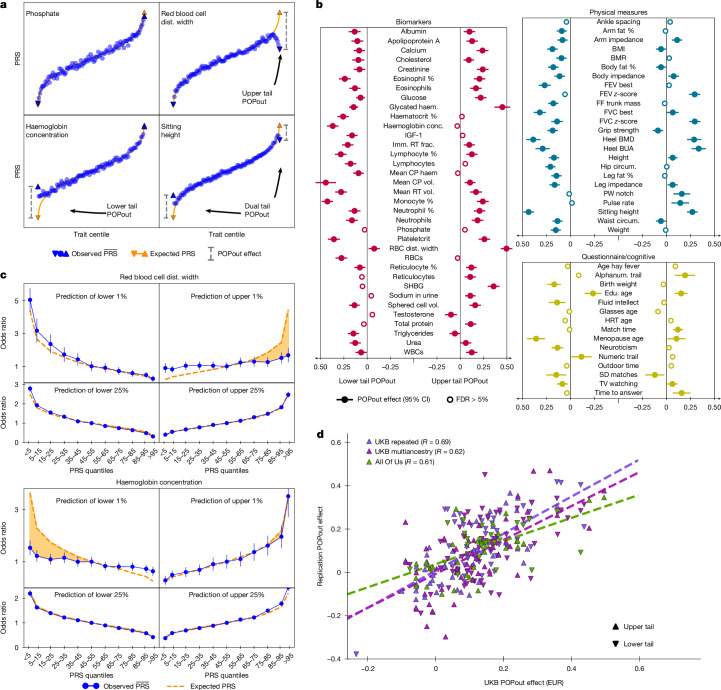
Population-based POPout analyses. **a**, Trait centiles plotted against mean PRS value for four traits with different types of POPout effect. Expected PRS (orange) inferred from regression of PRS on trait (Methods). **b**, POPout effect sizes with 95% confidence intervals are shown for the 74 traits of the primary analysis, calculated using two-sided *t*-tests (Methods) with a mean of *n* = 127,083 UKB individuals of European ancestry per trait. **c**, Odds ratios (with 95% confidence intervals) calculated in *n* = 129,100 (red blood cell distribution width) and *n* = 129,416 (haemoglobin concentration) individuals from logistic regressions of a trait-related hypothetical disease on PRS quantile (index quantile versus central quantile), with disease defined as the lower or upper 1% (or 25%) tail (controls otherwise). The orange dashed line indicates expected performance assuming a linear relationship between the PRS and trait (the shaded area highlights the difference between expected and observed). **d**, POPout effect sizes from UKB European ancestry analysis (from **b**) plotted against POPout effect sizes in the replication cohorts for upper and lower tails of all overlapping traits that passed QC. Pearson correlation coefficients between UKB European ancestry cohort and the replication effect sizes of the 50 traits from the UKB repeated cohort (*P* = 1.26 × 10^−15^; mean *n* = 12,007 independent samples per trait), 55 traits from the UKB multiancestry cohort (*P* = 3.63 × 10^−^^13^; mean *n* = 17,407 independent samples per trait) and 27 traits from the All of Us cohort (*P* = 1.07 × 10^−6^; mean *n* = 53,196 independent samples per trait) are shown (Methods). Alphanum., alphanumeric; BMI, body mass index; BMD, bone mass density; BMR, basal metabolic rate; BUA, broadband ultrasound attenuation; circum., circumference; conc., concentration; CP, corpuscular; dist., distribution; edu., education; EUR, European ancestry; FEV, forced expiratory volume; FDR, false discovery rate; frac., fraction; FF, fat fraction; FVC, forced vital capacity; haem., haemoglobin; HRT, hormone replacement therapy; imm., immature; PW, pulse wave; RBCs, red blood cells; RT, reticulocyte; SD, symbol digit; vol., volume; WBCs, white blood cells. Source data

Four ‘index traits’ provided examples of the observed POPout effects (Fig. 2a). Phosphate closely followed linear expectations, with negligible POPout effects. By contrast, red blood cell distribution width exhibited a pronounced upper tail effect (effect size of approximately 0.50 *Z* units), whereas haemoglobin concentration exhibited a pronounced lower tail POPout effect (approximately 0.37). Sitting height showed strong effects in both tails, consistent with symmetric enrichment of rare alleles expected under stabilizing selection. These four index traits were selected because they provided clear illustrations of different types of relationship; they are not representative of the general results.

Across the 74 traits, POPout showed widespread and often substantial departures from linear expectations in one or both tails (Fig. 2b). Sixty-eight traits showed significant POPout effects (false discovery rate < 5%) in at least one tail, most of which were positive effects, consistent with regression to the mean of the common PRS in extremes. In total, 108 trait tails were significant, with 98 showing positive POPout effects. This pattern of tail-specific architecture spanned trait domains, including blood cell indices, metabolic biomarkers, anthropometric measures, and behavioural and cognitive traits. The ten significant negative POPout effects could have resulted from epistatic, dominant or gene-by-environment effects, as more effect alleles than expected were associated with extreme values of these traits.

**Extended Data Fig. 1:**
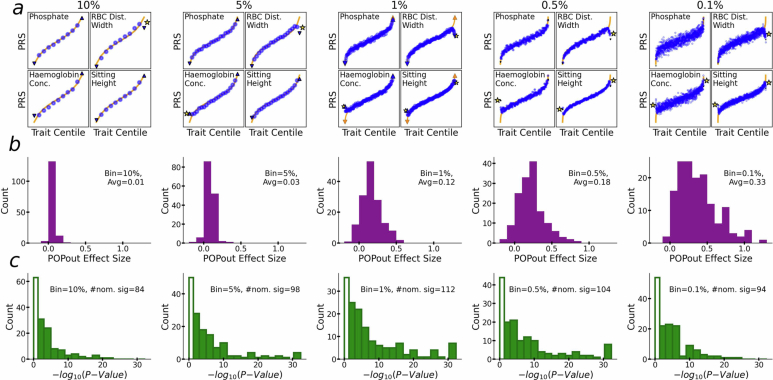
Sensitivity analyses of alternative POPout tail thresholds. **a**, Index trait PRS-on-trait plots for different POPout tail thresholds for testing. Nominally significant (*P* < 0.05) POPout effects are denoted by “*”. For tail sizes greater than 1%, POPout effects are reduced. At 10%, only red blood cell distribution width shows significance. **b**, POPout effect size distributions at different tail thresholds for testing. More liberal tail thresholds show reduced POPout effect sizes (Avg = mean POPout effect size). **c**, POPout *P*-value distributions (two-sided *t*-tests) at different tail thresholds for testing (white bar shows number of non-significant trait tails). The number of significant *P*-values is highest at 1%, reflecting a trade-off between POPout effect size (greater for more extreme tail thresholds) and statistical power (greater for more liberal thresholds). Source data

Sensitivity analyses across tail thresholds showed a consistent gradient (Extended Data Fig. 1). At 10% and 5% thresholds, fewer tails showed significant effects, and effect sizes were smaller (for example, mean effects of approximately 0.01 at 10%). At 0.5% and 0.1% thresholds, mean effects were larger (for example, approximately 0.18 at 0.5% and 0.33 at 0.1%), but fewer traits reached significance owing to reduced power. This suggests a biologically meaningful pattern, in which individuals whose trait value was driven by rare variants of large effect that were not captured by common PRSs, were increasingly likely to be found deeper in the tail.

## Reduced PRS accuracy

**Extended Data Fig. 2:**
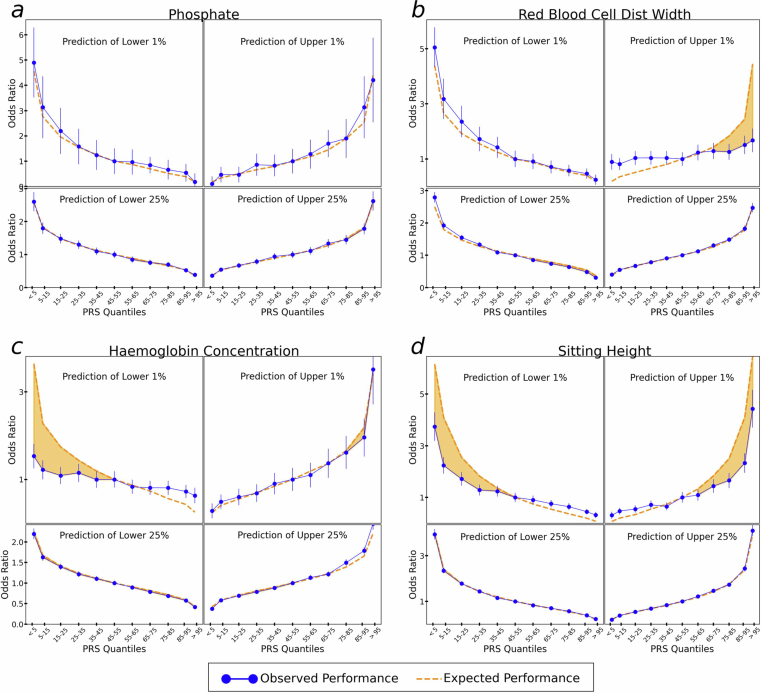
Impact of POPout effects on prediction for the four index traits. These results extend the analyses presented in Fig. 2c to the other two index traits (all four shown here). Odds ratios (with 95% CIs) from logistic regressions of a trait-related hypothetical disease on PRS quantile (index quantile vs central quantile), with disease defined as the lower or upper 1% (or 25%) tail (controls otherwise). Orange dashed line is expected performance assuming a linear relationship between the PRS and trait (shaded area highlights the difference between expected and observed). Source data

POPout effects imply that common-variant PRSs often underperform in prediction of phenotypic extremes, and that the accuracy of PRSs for diseases underlain by phenotypic extremes will be likewise limited. To illustrate this, we emulated PRS prediction of hypothetical diseases defined by different phenotypic thresholds in the index traits (Fig.2c and Extended Data Fig. 2). The PRSs for red blood cell distribution width and haemoglobin concentration performed well when disease was defined by the lowest or highest quartile, but they underperformed markedly when disease was defined by the lowest or highest 1%. For red blood cell distribution width, the mean odds ratio for individuals with the top 5% PRS values was expected to be 4.1 according to a linear trait–PRS relationship, but it was in fact 1.8; for haemoglobin, the expected odds ratio in the lower tail was 3.7, whereas the observed value was 1.8. These gaps reflect what POPout quantifies: when the PRS regresses towards the mean in the tails, the distinction between cases (extreme individuals) and controls weakens, limiting prediction at the highest-risk end. Thus, even when common PRS distinguishes between individuals of modest risk well, it can have poor performance in exactly those individuals at the highest risk of disease and most severe outcomes, such as early-onset disease and rapid progression.

## Replication of POPout effects

**Extended Data Fig. 3:**
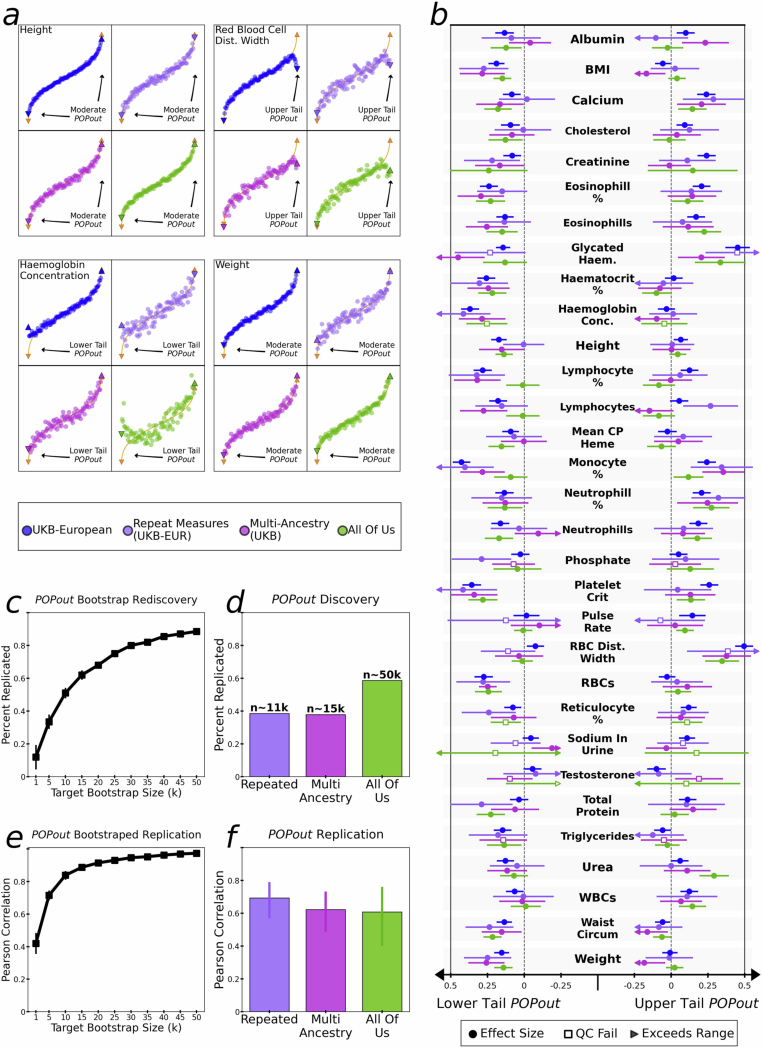
Replication of POPout effects. **a**, Trait centiles plotted against mean PRS value for the four index traits in the UKB-European (primary) analyses and in the three replication cohorts. Expected PRS (orange) inferred from regression of PRS on trait (Methods). **b**, POPout effect sizes (with 95% CIs) in primary and replication cohorts shown across the 31 overlapping traits of the All of Us cohort to allow trait-specific visualisation of replication results (shown in Fig. 2d) across all four cohorts (traits that failed QC in any cohort are highlighted). **c**,**d**, Considering trait tails with significant POPout results in the large European ancestry discovery analysis as true positives, the percentage of tails nominally replicated (*P* < 0.05) is shown across 100 bootstrapped runs of different sizes (c) and in replication datasets (d). **e**,**f**, Replication here is defined as a significant Pearson correlation (two-sided test) across effect sizes in bootstrapped subsets (e) and replication datasets (f). Source data

Here we performed three different forms of replication to test the generalizability of our primary findings, using our pretrained PRSs for each trait (Fig. 2d and Extended Data Fig. 3). In each replication analysis, which used samples independent of our primary analysis samples, we included only traits that passed our QC and had data for more than 5,000 individuals given inferred replication power (Methods). First, we tested UKB participants with repeated measures collected at baseline and follow-up (mean *n* = 12,007 individuals per trait). Averaging measures reduces the contribution of transient exposures such as short-term illness and medication use, as well as measurement error. POPout effect sizes in the repeated measures sample were strongly correlated with those of the primary analysis (*R* ≈ 0.69), suggesting robustness to transient environmental factors and measurement error.

Second, we performed a UKB replication in a held-out multiancestry sample (mean *n* = 17,407 individuals per trait). Despite the known challenges of PRS portability^22,23^, POPout effects were again strongly correlated with the primary analysis (*R* ≈ 0.62), indicating that tail deviations are a cross-ancestry phenomenon.

Third, we performed a matched-ancestry replication in individuals of European ancestry from the US-based All of Us cohort^24^ (mean *n* = 53,196 individuals per trait). This provided a stringent replication, because the cohort was recruited in a different healthcare setting on a different continent, using independent sampling, clinical and assay procedures. POPout effects were replicated with strong correlation (*R* ≈ 0.61) across overlapping traits. Together, these replications suggest that tail-specific departure from a linear trait–PRS relationship may be a stable and global feature of complex traits.

## Tail architecture inferred from siblings

Whereas the population-based POPout method relies on genotype data and PRS construction, family-based analyses offer an orthogonal approach that is robust to several forms of population structure^2^. UKB includes more than 17,000 sibling pairs of European ancestry not used in our population analyses. We therefore applied STANDout, a joint test that aggregates evidence from sibling-based models designed to distinguish tail architectures driven by common variants, by rare variants or by de novo mutations (Methods).

**Fig. 3:**
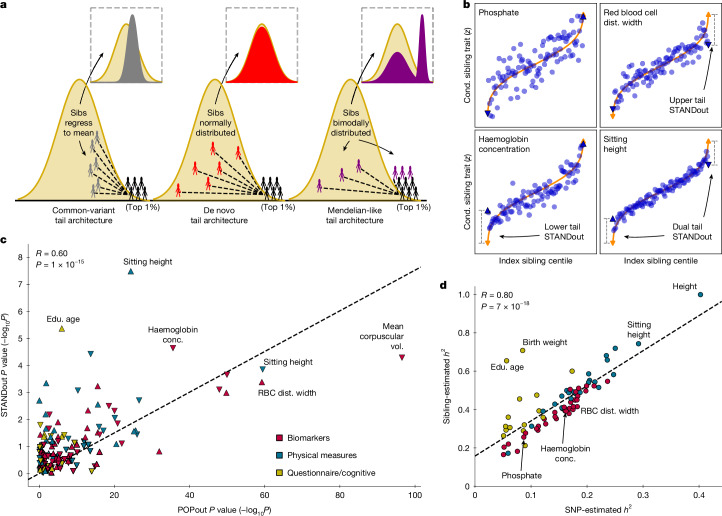
Tail architecture inferred from siblings. **a**, Schematic of relationships between sibling trait values under tails governed by common-variant (grey), de novo (red) and Mendelian-like (purple) architecture; here Mendelian-like refers to rare (but not de novo) large-effect alleles segregating in families. **b**, Index sibling trait centiles plotted against mean trait value of other (conditional) sibling for the four index traits (Fig. 2a). **c**, POPout *P* values (based on two-sided *t*-tests) plotted against STANDout *P* values (based on *χ*^2^ statistics with 4 d.f.) for the 74 traits. Pearson correlations and corresponding *P* values (two-sided test) are shown. **d**, LD score regression^25^ SNP-estimated heritability plotted against heritability estimated from sibling-pair trait similarity (Methods). Pearson correlations and corresponding *P* values (two-sided test) are shown. Sibs, siblings. Source data

The key intuition is that differences among siblings for a trait are partly governed by the genetic architecture of the trait (Fig. 3a). Under common-variant architecture, siblings of individuals with extreme trait values are expected to have high but less extreme trait values, depending on heritability. Under de novo-dominated tail architecture, siblings resemble the population background, whereas under Mendelian-like tail architecture driven by large-effect segregating rare alleles, half the siblings share the large-effect allele and also have extreme trait values, whereas the other half resemble the population. STANDout combines evidence across these patterns to test for departures from common-variant expectations.

**Extended Data Fig. 4:**
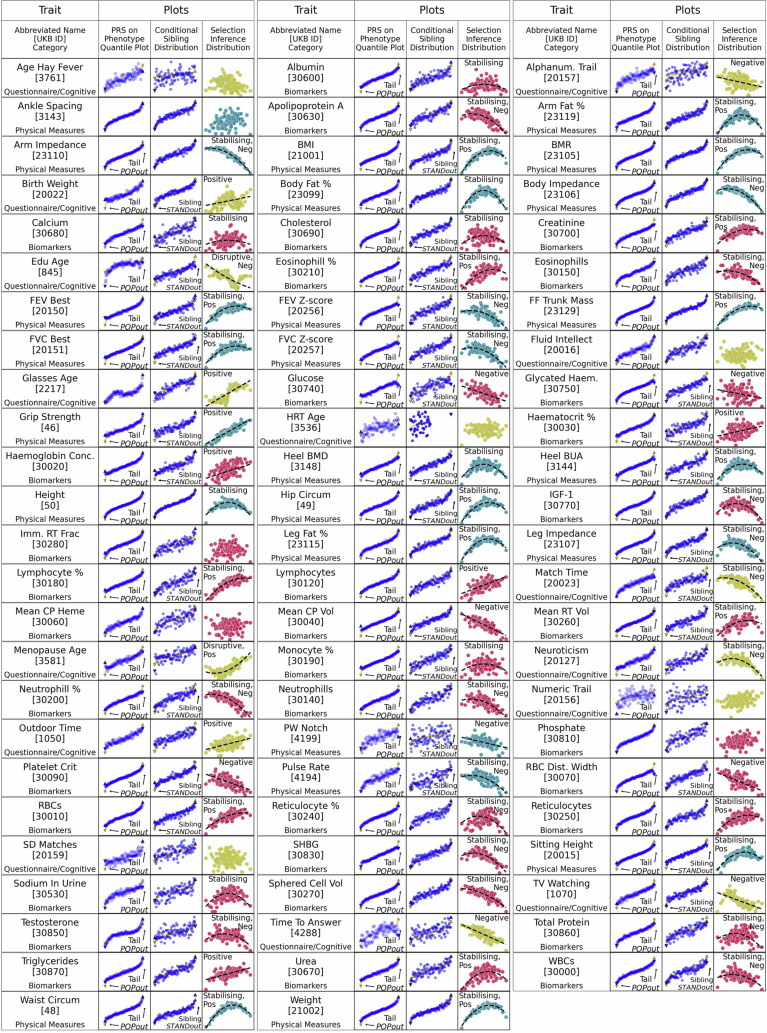
Result summaries for all 74 analysed traits. Comprises PRS-on-trait quantile plots, conditional sibling distributions and selection inference distributions. Source data

Across the 74 traits, STANDout results broadly replicated the POPout results. In terms of the index traits, STANDout recapitulated the lack of tail deviation for phosphate and identified strong tail-specific deviations from common-variant architecture for haemoglobin concentration, red blood cell distribution width and sitting height (Fig. 3b). Overall, POPout and STANDout results were highly correlated (*R* ≈ 0.60; Fig. 3c and Extended Data Fig. 4), indicating that two largely orthogonal data sources—population genotype-based PRSs versus family phenotypes—converge on the same conclusion; that is, that many trait extremes are consistent with departures from common-variant architecture. Further support for the validity of this sibling-based approach was provided by the strong correlation (*R* ≈ 0.80) between single nucleotide polymorphism (SNP)-based heritability^25^ and sibling-based heritability (Fig. 3d), which is estimated as a key parameter in the STANDout method.

## The role of rare variants

**Fig. 4:**
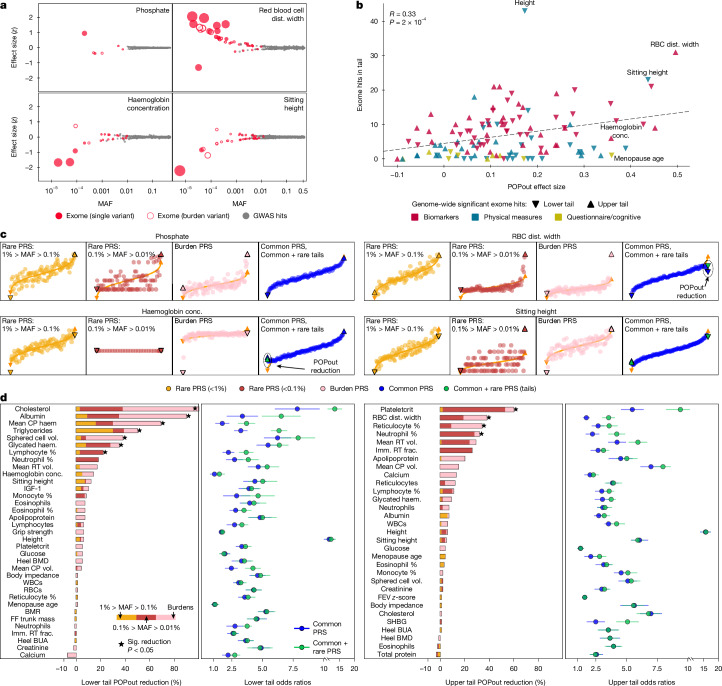
The role of rare variants in observed POPout effects of the primary analysis. **a**, Trumpet plots^4^ (MAF versus effect size) of significant variants reported in major exome^26^ and GWAS^40^ analyses for the four index traits (Fig. 2a). **b**, POPout effect size versus number of significant exome variants^26^. For upper tail POPout, the number of trait-increasing rare alleles was counted; for the lower tail, trait-reducing alleles were counted. Pearson correlations and *P* values (two-sided test) are shown. **c**, PRS-on-trait centile plots for three rare PRSs, the common PRS and the rare + common PRS (shown in tails) for the four index traits. **d**, POPout effect size reduction (left), calculated as a percentage of original POPout effect size. Nominally significant *P* values for one-sided paired *t*-tests for POPout reduction in 11 traits: cholesterol (*P* = 3.5 × 10^−5^), albumin (*P* = 6.2 × 10^−^^3^), mean CP haem (*P* = 8.2 × 10^−^^3^), triglycerides (*P* = 0.024), sphered cell volume (*P* = 0.036), glycated haemoglobin (*P* = 0.04), lymphocyte percentage (*P* = 0.014), plateletcrit (*P* = 6.9 × 10^−5^), RBC distribution width (*P* = 2.1 × 10^−^^6^), reticulocyte percentage (*P* = 0.037) and neutrophil percentage (*P* = 0.043). Tail odds ratios (right) before and after addition of WGS and WES rare variants to common PRS are reported. Odds ratios (with 95% CIs) were computed in a mean of *n* = 128,571 individuals from logistic regressions of a trait-related hypothetical disease on PRS quantile (lower or upper 1% PRS versus remaining 99%), with disease defined as the lower (left panel) or upper (right panel) 1% tail (controls otherwise). Source data

Although POPout and STANDout infer tail-specific departures from common-variant architecture consistent with rare-variant enrichment, they do not themselves identify rare variants. Here we sought direct evidence for the role of rare variants. First, we leveraged published results from UKB exome analyses^26^ to test whether traits with larger POPout effects harboured more significant rare coding associations (Fig. 4a,b). For each trait and tail, we counted the number of significant exome ‘hits’ (single-variant or burden signals) with effects in the direction implied by the POPout effect. Across traits, POPout effect size was correlated with the number of such exome hits (*R* ≈ 0.33), linking tail deviations to rare-variant association evidence.

Second, we incorporated rare variants directly using UKB WES and WGS data in the subset of individuals with genotype array, WES and WGS available (more than 99% of the primary analysis sample was retained)^26,27^. We included traits with at least one significant positive POPout effect and constructed three rare-variant PRSs for each trait: two based on single-variant associations from WGS, stratified into moderately rare (0.1% < MAF < 1%) and very rare (0.01% < MAF < 0.1%) components; and a third based on gene-burden signals from WES (Methods). We then combined these with the common-variant PRS to create a ‘rare + common’ PRS and reassessed POPout effects. Although these variants were identified in only half the UKB sample, they were highly represented in the ClinVar database (Extended Data Tables 1–3). For example, significant single-variant and burden signals were linked to well-established disease genes present in the ClinVar database^28^, including *ACAN*, *HBB*, *JAK2*, *LDLR*, *MC4R*, *TFR2* and *CHEK2*.

For many traits, rare-variant PRSs showed tail-localized ‘spikes’ that coincided with trait tails where POPout effects were observed, and incorporating these rare PRSs shifted tail PRS values towards linear expectations (Fig. 4c). Across 98 trait tails with significant positive POPout effects, 32 corresponded to traits with no significant rare variant identified, whereas 56 of the remaining 66 tails showed POPout reductions (Fig. 4d). Of those, 11 had significant and substantial reduction in POPout effects, with a mean reduction of around 50%. The lower tail effects included elimination of the POPout effect for cholesterol, a 90% POPout reduction in albumin and a 34% reduction in glycated haemoglobin, whereas for upper tail effects there was a 60% reduction in POPout effect for plateletcrit and a 38% reduction for red blood cell distribution width.

Overall, burden variants were responsible for the largest reductions in POPout effects, consistent with results from our population genetic simulations and empirical evidence of larger contributions to rare-variant heritability from ultrarare variants^29^. Therefore, much of the residual POPout effects could be explained by ultrarare variants that are yet to be identified or have effect sizes that are not well captured by their crude inference from burden tests.

To illustrate the potential impact of these POPout reductions on disease prediction, we calculated odds ratios for PRS prediction of a hypothetical disease defined as corresponding to the lower or upper 1% tail, for each trait tail before and after inclusion of rare variants (Fig. 4d and Methods). Although the impact of the rare variants on PRS *R*^2^ was modest (mean relative *R*^2^ increase of 11.6%), their impact on tail-specific PRS odds ratios was substantial (mean relative odds ratio increase of 71.7%). This highlights how rare variants can be clinically and predictively critical even when they explain little variance at the population level.

**Extended Data Fig. 5:**
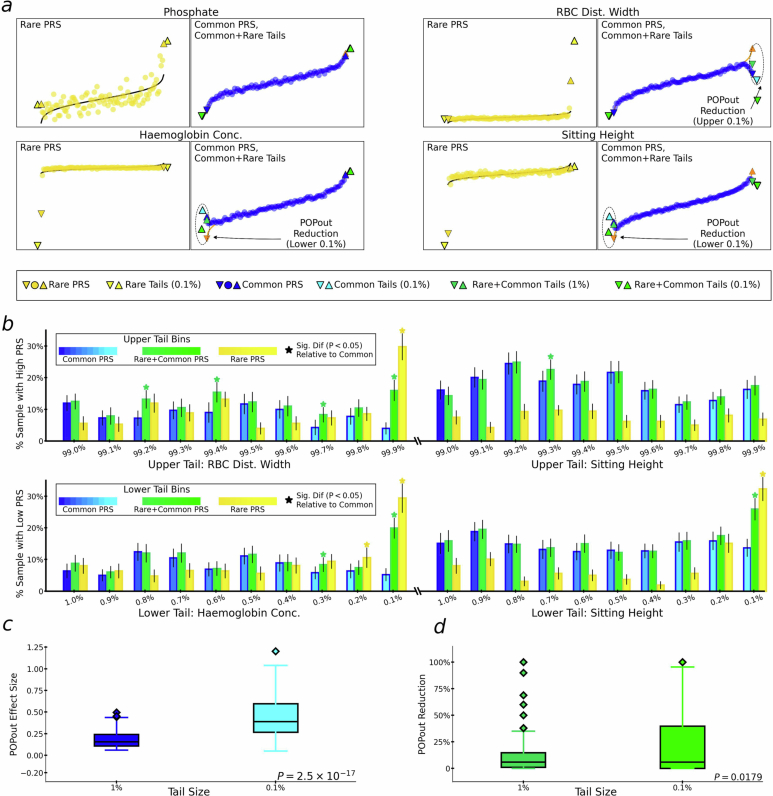
The role of rare variants in POPout effects of the primary analysis deeper into the trait tail. **a**, PRS-on-trait centile plots for the rare PRS (combining the three rare PRSs of Fig. 4c), the common PRS, and the common+rare PRS for the four index traits, with tail effects shown for the 1% tails as well as the 0.1% tails. **b**, The percentage of individuals with high (/low) PRS in 0.1% bins of the upper (/lower) tail, in which high (/low) PRS is defined as being in the highest (/lowest) 5% of PRS, for the four index traits **c**, POPout effect sizes in relation to the 98 trait tails with positive, significant (FDR < 0.05) common-variant PRS POPout, shown when computed in the 1% tail and the 0.1% tail (box-and-whiskers correspond to median, interquartile range, 1.5* interquartile range and outliers). *P*-value shown corresponds to the result of a two-sided *t*-test for the difference in POPout effects between the 1% and 0.1% tails, **d**, Differences in POPout reduction in the 1% and 0.1% trait tails, shown in relation to the 66 trait tails with positive, significant common-variant POPout and at least one genome-wide significant rare variant (box-and-whisker components as in **c**). *P*-value shown corresponds to the result of a two-sided *t*-test for the difference in POPout reduction between the 1% and 0.1% tails. Source data

The contribution of rare variants increased deeper into the tails (Extended Data Fig. 5). At 0.1% thresholds, POPout effect sizes were larger, as were POPout reductions after inclusion of rare variants, but there was also greater residual POPout to explain versus the 1% tails. This pattern is consistent with an increasing role of ultrarare variants with very large effects among the most extreme individuals, as many of these variants are likely to be beyond current discovery power even in large biobanks and may also include non-coding and structural variation.

## The role of natural selection

**Fig. 5:**
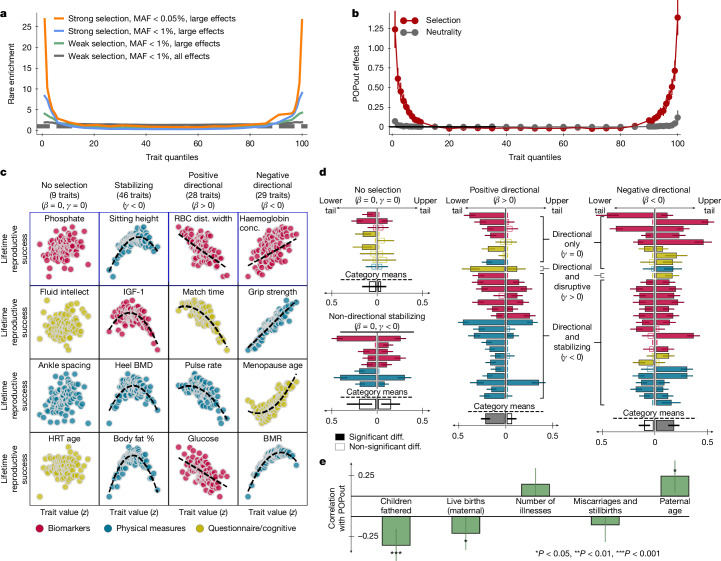
The role of natural selection in observed POPout effects. **a**, Enrichment of rare vs common alleles in SLiM-4 (ref. ^17^) simulations under weak and strong selection scenarios, stratified by rare-variant classification and effect size. **b**, POPout effects across trait quantiles averaged over 100 simulations (mean values and 95% CI are shown) under neutrality and ‘weak’ selection. **c**, Trait value versus relative lifetime reproductive success for 16 example traits. The best-fitting model (dashed line) determined by BIC (*β*, linear; *γ*, quadratic; regression parameters) was used to classify selective pressure acting on traits. **d**, POPout effect sizes (with 95% CIs) for traits are grouped by selection category inferred by BIC model (models with linear and quadratic terms are categorized by direction of linear term). Differences between lower and upper tail effects were tested by two-sided *t*-tests in each of the four categories. Negative POPout effects are shown as having POPout effects of 0 (without CIs shown) for illustrative convenience; testing was performed on the actual POPout effects. **e**, Association between POPout effect size and five traits relating to fitness. For each fitness trait, we show the Spearman’s rank correlation (with 95% CI) between the mean value of the fitness trait in individuals in the upper and lower tails of each of the 74 traits and the corresponding POPout effect size (calculated across 148 pairs of values), with two-sided nominal *P* values: children fathered (*P* = 5.9 × 10^−^^4^), live births (maternal) (*P* = 0.048), number of illnesses (*P* = 0.16), miscarriages and stillbirths (*P* = 0.32) and paternal age (*P* = 0.018). Source data

To test whether selection generated POPout-like signals, we performed forward-in-time simulations using SLiM-4 (ref. ^17^). We simulated polygenic traits with effect sizes drawn from a gamma distribution and compared neutrality with periods of stabilizing selection of varying strength (Methods). After stabilizing selection, rare alleles of large effect were enriched in the trait tails compared with under neutrality, and this effect was more pronounced for very rare alleles and after strong stabilizing selection (Fig. 5a). Under neutrality, approximately 1% of simulated individuals in the lower and upper trait centiles harboured rare alleles of sufficiently large effect to cause their extreme trait value, whereas around 28% of individuals in the tails carried such alleles under strong stabilizing selection. Moreover, under stabilizing selection, the relationship between phenotype and common PRS became nonlinear in the tails (Fig. 5b), mimicking the POPout effects of our real data analyses.

**Extended Data Fig. 6:**
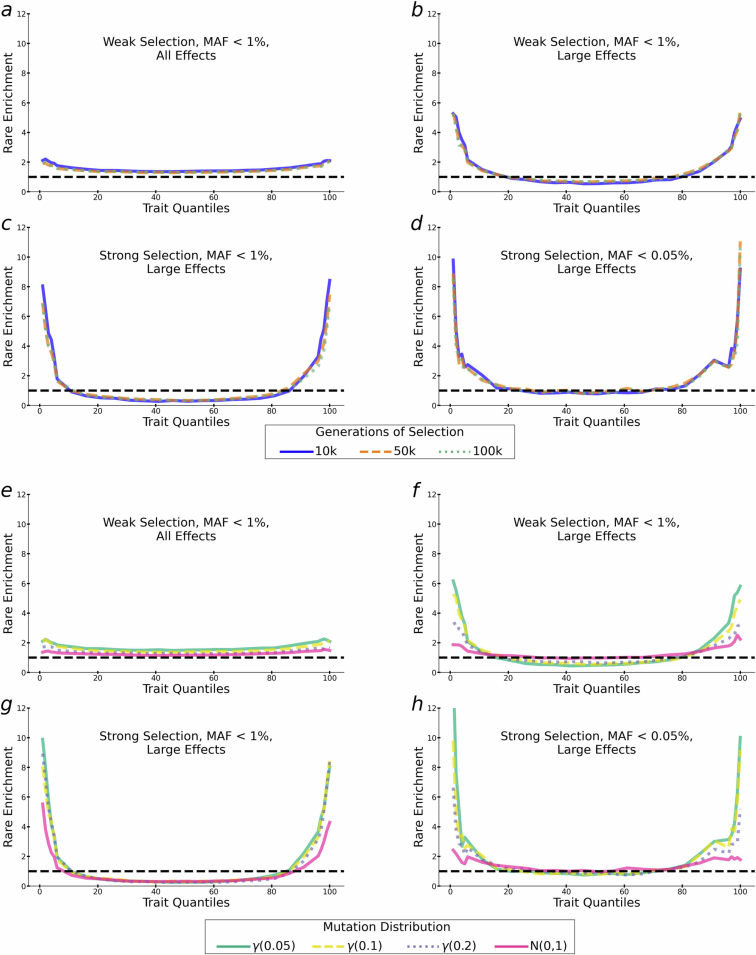
Enrichment of rare vs common alleles in forward simulations under different numbers of generations and effect size distributions. Enrichment of rare vs common alleles in SLIM-4^17^ simulations (Methods) under weak and strong selection scenarios, stratified by rare variant classification and effect size, shown for (a-d) different durations in terms of numbers of generation of the simulated population, and shown for (**e-h**) different effect size generating distributions (gamma distributions with different means and Gaussian) of mutations. Source data

**Extended Data Fig. 7:**
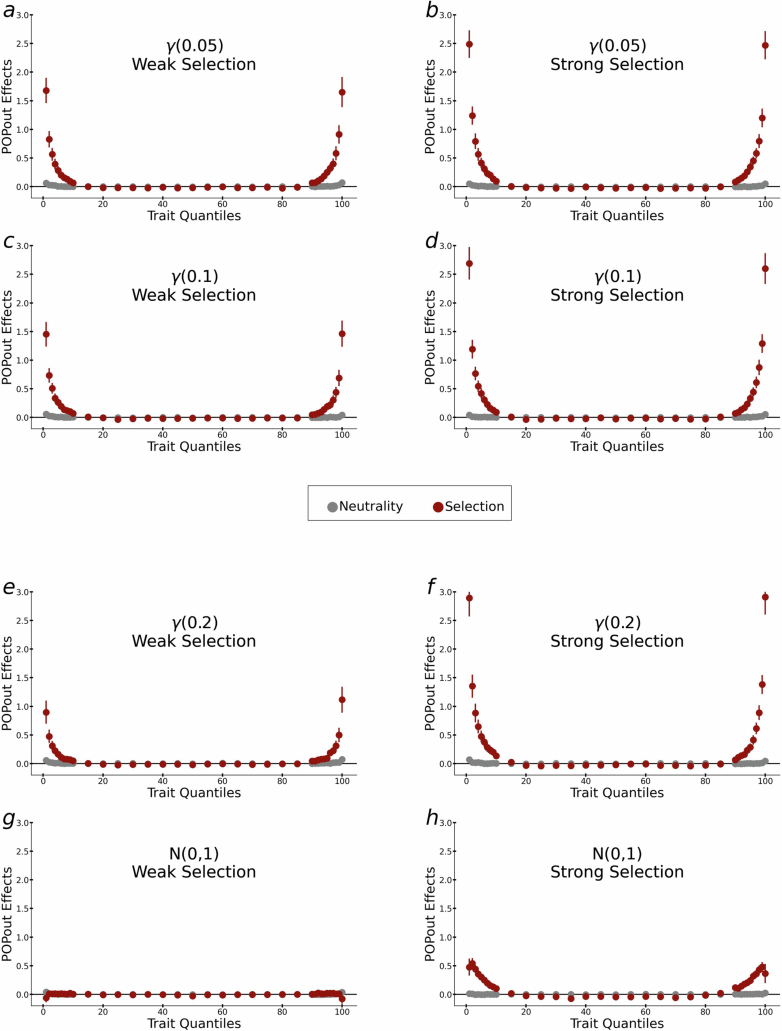
POPout effects under different effect size distributions and strengths of selection. POPout effects across trait quantiles averaged over 100 SLIM-4^17^ simulations (Methods) under weak and strong selection (mean and 95% CIs shown), stratified according to different effect size generating distributions of mutations. Source data

The results were highly similar for sensitivity analyses in which the period of selection was extended and when different gamma distributions for the effect sizes of mutations were used (Extended Data Figs. 6 and 7). Rare-variant enrichment and POPout effects were markedly diminished when a Gaussian distribution was used for mutational effect sizes, suggesting that a more realistic heavy-tailed distribution, such as the gamma distribution, may be required to explain the observed POPout effects^30^. These simulations suggest that selection can have a critical impact on how genetic architecture varies across the trait continuum and can generate substantial rare-variant architecture in the tails of traits that are otherwise governed by common-variant architecture.

Next, we investigated empirical evidence for natural selection as a source of observed POPout effects. It is challenging to infer selection acting on traits from contemporary data, especially in a way that is not inherently correlated with POPout effects through reliance on shared genetic signatures. However, Sanjak et al.^16^ have developed a method that infers continuing selection from non-genetic data by modelling the relationship between trait values and lifetime reproductive success in a regression framework. Here we extended this approach (Methods), applying model selection across linear and quadratic terms to classify traits into categories consistent with neutrality, non-directional stabilizing selection, and positive or negative directional selection (Fig. 5c), with the latter two potentially incorporating a stabilizing or disruptive selection component.

We proposed that traits inferred as having no selection would have no POPout effects, those with non-directional stabilizing selection would have similar lower and upper tail POPout, those inferred as under positive directional selection would have larger lower tail POPout effects on average, and those under negative directional selection would have larger upper tail POPout effects. To test this hypothesis, we stratified the POPout results from the primary analysis into groups of traits according to the categories of inferred selection and determined whether the POPout effects reflected the inferred selection (Fig. 5d). There were no significant differences in mean POPout effect between lower and upper tails that were inferred to be under no selection (*P* = 0.25) or non-directional stabilizing selection (*P* = 0.52). By contrast, there were significantly larger POPout effects in the lower tail for traits with inferred positive directional selection (lower mean = 0.20, upper mean = 0.049, *P* = 2.9 × 10^−6^) and significantly larger POPout effects in the upper tail for traits with inferred negative directional selection (lower mean = 0.085, upper mean = 0.17, *P* = 0.014).

We verified the robustness of our results in two ways. First, we confirmed that our inferences of different types of selection were aligned with those by Sanjak et al. for the traits that overlapped between studies^16^ (Supplementary Table 1); second, we confirmed that selection of the second-best Bayesian information criterion (BIC) model, when it was not significantly different from the best model, produced similar results (Supplementary Fig. 1). Despite the limited snapshot into historical selection provided by contemporary reproductive success, which can be influenced by social structure and reverse causation, the concordance of POPout patterns with inferred selection observed here supported the contribution of selection to tail architecture.

Finally, we tested for associations between POPout effects of the 74 traits of the primary analysis and measures of fitness available in the UKB: number of offspring, miscarriages and stillbirths, paternal age and self-reported illness (Fig. 5e). For each fitness measure, Spearman’s rank correlations between the mean fitness value of individuals in each trait tail and the corresponding POPout effect were computed (Methods). POPout effects were significantly negatively correlated with number of offspring (paternal: *P* = 5.9 × 10^−^^4^; maternal: *P* = 0.048), significantly positively correlated with paternal age (*P* = 0.018) and not associated with self-identified illnesses or number of miscarriages and stillbirths. The significant reduction in number of offspring was consistent with tails that have POPout effects being subject to selective constraint, whereas advanced paternal age has been associated with higher rates of disorders in offspring and reduced fitness due to increased de novo mutation rates^31^.

## Discussion

Applying population-based PRS method POPout and family-based method STANDout, we uncovered widespread departures from common-variant architecture in the tails of a diverse range of quantitative traits. Our replication results indicate that these tail deviations may be a stable, global phenomenon. Although we expect future work to further identify non-genetic factors contributing to these patterns, here we established large-effect rare alleles as a major determinant. Forward simulations demonstrated that stabilizing selection drives these alleles to the trait tails, as it generates a negative exponential relationship between MAF and effect size and reduces trait variance^4,6,17^. Empirical analyses based on lifetime reproductive success showed that POPout patterns are aligned with inferred direction and type of contemporary selection across traits^16^. Together, these findings provide robust support for our overarching hypothesis: namely, that selective pressures on many complex traits have led to distinct concentrations of high-effect rare alleles in their tails. Therefore, although complex traits are broadly polygenic in the population, they often become oligogenic in one or both of their tails, depending on selective pressures on the trait.

These findings have several practical and theoretical implications. First, they motivate a general strategy for rare-variant discovery: prioritize for sequencing—or upweight in rare-variant and gene burden^32^ testing—individuals whose extreme phenotype is poorly explained by common PRS. Application of POPout can indicate the potential yield of such a design for each trait tail. In addition, integrating family information can help to distinguish segregating versus de novo contributions, guiding the choice between population sequencing and trio-based designs as the optimal approach^2^. We provide user-friendly software to implement the POPout and STANDout tests, which are applicable to widely available large-scale population genotype and family trait data. Second, POPout effects highlight theoretical limits on common-variant PRS. Incorporating rare variants into PRS will improve prediction, as demonstrated here and elsewhere^10^, but if much of the missing trait variance in the tails is explained by ultrarare variants that are hard to identify or have effects that are hard to accurately infer, prediction will remain limited. However, iterative application of our approaches can help to overcome this: the approaches can be first used to increase rare-variant discovery; then, newly discovered rare variants can be integrated into PRS, increasing PRS accuracy and, in turn, the power of subsequent POPout analyses to identify previously unidentified rare variants. Third, our results indicate a potential framework for integration of common and rare variants in prediction. Common-variant PRS captures broad liability across the distribution, but prediction of the most clinically relevant extremes with POPout effects may require a dedicated effort to boost the identification of rare alleles. Even when the incremental variance explained by rare variants is modest, the contribution of these variants to risk stratification can be large, as reflected by increases in tail-specific odds ratios after incorporation of rare variants (Fig. 4).

Tail-specific architecture also suggests consequences for PRS portability: (1) differences in diagnostic thresholds of a disease in different populations could result in a disease being underlain by a different relative mix of common and rare variants, causing variable performance of common-variant PRSs across populations; and (2) rare alleles are often geographically localized and more sensitive to demographic factors than common variants^33^. Therefore, as rare variants are incorporated into PRS to improve tail prediction, portability across populations may decrease, particularly for traits subject to local selective pressures^34,35^. Our multiancestry replication indicates that tail deviations may often be consistent across ancestries, but the exact rare variants that contribute to extremes may differ.

Finally, we offer a new perspective on how selection shapes human genetic variation, through its impact on how genetic architecture varies along the trait continuum. Recent work has led to important advances in our understanding of how historical selection may have generated and can be inferred from widespread polygenicity^6,36^. Our work may help to build on this by motivating new ways to improve inference of selection, for example, by evaluating whether asymmetric POPout effects suggest recent shifts from stabilizing to directional selection towards a new optimal trait value^13^, or through modelling the effect sizes of rare variants that are unobserved but informative. Further modelling of the causes of POPout effects may also provide insights into mutational target sizes of traits^37^, especially the component of the mutational target corresponding to large effects^38^.

Our study has several limitations and areas for future follow-up. First, and most important, despite our focus on genetic causes, we expect environmental factors and technical factors such as trait skewness and measurement error to have contributed to our observations. However, given control of major covariates, analysis of traits with limited skew, replication across repeated measures and populations, reduced POPout effects after inclusion of rare variants, and evidence of selection shaping tail architecture, we believe that high-effect, rare alleles are a major cause of our findings. Trait-specific follow-up will be important to disentangle genetic and non-genetic drivers for individual phenotypes. Second, our multiancestry replication combined individuals of diverse ancestries into a single group, which poorly reflects global diversity and precludes evaluation of heterogeneity. However, large sample sizes are required to study trait-tail architecture, and so we performed this grouping to establish whether our findings were valid across ancestries. Future studies performing these analyses in large ancestry-specific cohorts will be better powered to characterize tail architecture across global populations.

Our rare-variant analyses also remain incomplete. We focused on rare single variants and coding burden signals that are currently most tractable, but trait extremes may also be influenced by ultrarare variants below current discovery thresholds, structural variants, repeat expansions and non-coding regulatory variation with large effects. The residual POPout signal in the deepest tails suggests that a substantial component of rare variation remains uncaptured. Increasing sequencing sample sizes, improving functional priors, and integrating family-based sequencing designs will help to address this gap. In addition, we did not consider pleiotropy among traits. Pleiotropy affects selective pressures on variants and thus their allele frequencies^14^, and it will have contributed to the widespread nature of the POPout effects, as indicated by variants included in our rare PRSs affecting multiple traits (Extended Data Tables 1–3). However, our findings and conclusions should be valid irrespective of pleiotropy.

We did not consider complex disease explicitly here, as our POPout method can be applied only to continuous outcomes. However, findings from disease studies are consistent with our observations^11,12,39^, and our POPout approach could be applied to a disease biomarker or risk factor instead, providing a new strategy for identification of rare variants affecting a subset of disease patients. Finally, selection was inferred empirically using fecundity data, which acts as a crude proxy of selective pressures on a trait and is vulnerable to reverse causation. Despite these limitations, we observed significant associations between selection inferred from fecundity and POPout effects.

Overall, our results establish a general trait-wide phenomenon: polygenic trait architecture shifts to a more heterogeneous, oligogenic form in one or both tails of complex traits owing to pervasive selective constraints on phenotypic extremes. Recognizing this shift provides a new perspective for interpretation of PRS performance, design of rare-variant discovery studies and understanding of how selection shapes the genetic basis of human phenotypes. It also highlights the point that rare variants can have a substantial impact on phenotypic extremes and severe disease despite their relatively low heritability. Future work could build on this study to explicitly disentangle the relative contributions of different genetic and environmental causes of extreme trait values and disease in individuals and populations.

## Methods

### Data processing

#### UKB genotype data

The UKB is a prospective cohort study of approximately 500,000 participants recruited across the United Kingdom from 2006 to 2010 (ref. ^18^). Phenotype data of anthropometric, biological and lifestyle measures were collected at baseline and in follow-up surveys, with further linkage to health and disease record data. The genetic dataset consists of 488,377 samples genotyped at 805,426 SNPs. To define population ancestries, 4-means clustering analysis was conducted on the first two principal components of the genotype data. Ancestries were then defined according to the country of birth (field ID: 20115) of the majority of individuals in the cluster, resulting in 461,931 European, 11,074 South Asian, 7,935 African, 2,585 West Asian and 2,550 East Asian individuals, and 1,619 individuals in clusters for which there was no majority country of birth.

Subsequent to clustering, standard QC procedures were applied independently to each ancestry cluster. SNPs with a minor allele frequency (MAF) < 0.01, genotype missingness > 0.02 or Hardy–Weinberg equilibrium test *P* < 10^−8^ were excluded. Samples exhibiting high levels of missingness or heterozygosity, or inconsistencies in genetic-inferred and self-reported sex, or showing aneuploidy of the sex chromosomes were removed in accordance with recommendations from the UKB data processing team^18^. For analyses in the general population, a greedy algorithm was used to remove related individuals to maximize sample retention while removing all third-degree relatives (kinship coefficient > 0.044)^41^. After these QC steps, 411,948 unrelated individuals remained, of whom 387,472 were of European ancestry. The European ancestry cluster included 18,340 individuals with repeated measures that were set aside, as well as 24,476 individuals of multiple non-European ancestries for replication analyses. This resulted in up to 369,132 unrelated individuals of European ancestry for the primary POPout analyses.

For the sibling analyses, sibling pairs were selected by first identifying first-degree relatives, defined as those with kinship coefficient within the range 0.177–0.354 (the expected value for siblings is 0.25) to account for expected variation among siblings^42^. Only individuals of European ancestry were included in the sibling analyses owing to insufficient statistical power of the multiple ancestries sample^2^. The proportion of SNPs with zero identity-by-state (IBS0) was used to distinguish sibling pairs from parent–offspring pairs, as parent–offspring pairs have IBS0 ≈ 0 across the autosomes. In the UKB, there is clear separation between pairs of first-degree relatives at IBS0 = 0.00112 (ref. ^42^); thus, sibling pairs were selected as those exceeding this threshold. This provided a total of 17,289 sibling pairs for analysis.

### Quantitative trait QC

#### Initial trait selection

We considered an initial list of 408 non-procedural continuous traits from the UKB categories of biomarkers, physical measures, the touchscreen questionnaire and cognitive function to provide a broad group of well-studied traits. We removed three traits related to lifetime reproductive success used as outcomes in the selection analyses to avoid circularity, as well as three predicted traits that had directly measured alternatives. To minimize non-genetic causes of trait values, we then removed all individuals with a cancer diagnosis (34,698) and those on insulin treatment (1,311) or who were pregnant at baseline (109). We also removed 64,043 individuals on statins and other cholesterol-lowering drugs from the blood count and blood biochemistry subcategories and 29,395 individuals on heart rate medication from the arterial stiffness and blood pressure subcategories. Next, to ensure that extreme outliers did not affect results, we removed all samples that had trait values six standard deviations or more from the mean. After removal of these samples, we removed traits for which the top two modal values comprised more than half the samples and traits with fewer than 100,000 samples remaining in our primary dataset of unrelated individuals of European ancestry, resulting in a set of 141 quantitative traits. These traits were then residualized and subjected to further QC.

#### Trait residualization

To minimize the impact of environmental risk factors, we residualized trait values within each ancestry group (European and multiancestry) by applying a linear regression model to each trait, with covariates for age, age^2^, age^3^, sex (as reported by the UK National Health Service at birth, unless since revised by the participant), menopause status (among females), type II diabetes status, coronary artery disease status, alcohol intake (field ID: 1558), smoking (categorical, field ID: 20116; continuous, field ID: 1239), batch, centre and the first 40 genetic principal components. At this stage, trait residuals with an absolute skew greater than 2 in the larger European ancestry group were removed to mitigate measurement bias, and the remaining trait values were standardized using a rank inverse normal transform on the residuals. This inverse normal transformation of the traits introduced a conservative bias in the POPout test, although this was expected to be small because of the removal of highly skewed traits and application to trait residuals, and was performed to prevent inflation of the statistic in the linear regression framework of the tests. This provided traits adjusted by key environmental factors and genetic principal components and with relatively low skew, transformed to have standard normal distributions for all subsequent analyses.

#### GWAS and further QC

For our initial primary analyses, European ancestry UKB samples were split randomly into 50% base and 50% target datasets, for computation of GWAS results and PRS, respectively. Then, for each trait, a GWAS was performed using the standardized trait values in the base data, applying PLINK-1.9 (refs. ^43,44^), whereas heritability and pairwise genetic correlations were estimated using LD score regression^45^. To ensure that our PRS analyses were well powered, we removed traits with heritability ${h}^{2} < 5 \%$ (ref. ^19^), and to ensure that the traits were essentially polygenic rather than oligogenic, traits were removed for which a single SNP (MAF > 1%) explained more than 2% of trait variance^46^. We also removed traits on the basis of deviations from trait–PRS linearity in the body of the trait distribution (‘POPout test QC’). After these QC steps, 114 quantitative traits remained across all four categories.

To ensure that the traits were different from each other, for traits with pairwise genetic correlation greater than 0.98, the trait with larger sample size was retained to maximize power for analyses; in addition, for correlated networks of traits, the traits that ensured optimal retention of traits were removed. This led to a further 36 traits being removed. A further four traits were removed on the basis of manual assessment of retained traits, as genetic correlation can be underestimated between traits with large differences in sample size. Together, these steps resulted in a set of 74 traits for use in the primary analyses. This broad set of traits ensured comprehensive coverage over a diverse set of phenotypes and provided adequate numbers of overlapping traits for the replication analyses.

Using scripts provided (‘Code availability’) researchers can perform this QC pipeline or change the QC thresholds to produce bespoke analyses, with information output on the traits that are removed and retained at each stage, and generate a full set of results.

### PRS calculation

The 74 traits remaining after QC comprised 35 traits in the biomarkers category, 24 traits in the physical measures category, and 15 that were in either the touchscreen questionnaire or the cognitive function category. In the primary unrelated European dataset, these traits had between 50,109 and 167,857 (mean 123,482) samples in each of the base and target datasets. For each (residualized) trait, PRSice-2 (ref. ^47^) was used to calculate PRSs for all individuals of the target data (second half of data), using base data GWAS (see above) and the FastScore option, which selects the optimal PRS from one of ten *P* value thresholds. Each PRS and corresponding trait was then used in the POPout analyses.

The POPout test was performed in the target sample, that is, the sample in which the PRSs were trained, to optimize sample size for testing in the tails. Although each PRS was fitted (by means of *P* value threshold optimization) to the same data used for POPout testing, the use of only ten *P* value thresholds was expected to minimize this overfitting to the target sample; this is a conservative bias in any case, because the PRSs were fitted assuming a linear relationship between the PRS and trait. None of the replication analyses (below) was subjected to any overfitting as they all corresponded to out-of-sample data.

### Replication datasets

#### European ancestry repeated measures

After initial QC of the UKB sample (above), there were up to 18,340 European ancestry individuals with measurements repeated at a follow-up data collection an average of approximately 5 years after baseline (timing differed for different individuals) for 63 of the 74 traits from the primary analysis. For these individuals, both measurements were normalized alongside the other European ancestry UKB samples, as described above, but held out of the base dataset. Then, the residualized trait values from their two visits were averaged (mean) to provide a target subset of individuals for whom random measurement error or transient effects (for example, infections) were less likely to produce trait values in the tail. For the 63 traits, there were between 2,461 and 15,475 unrelated individuals with repeated measures available for replication.

#### Multiancestry sample

After initial QC, there were up to 24,476 individuals of multiple ancestries (in order of sample size: South Asian, African, West Asian, East Asian and other smaller sized ancestries; see ‘UKB data’ section for classification of ancestries) available for replication; we refer to this sample as the multiancestry replication sample. The trait values of these individuals were normalized alongside those of the European UKB individuals, and PRSs were calculated for the 74 traits of our primary analyses using PRSice-2 (ref. ^47^). PRSs were calculated in this sample using the *P* value threshold (and thus the same SNPs) optimized for the relevant trait in the primary analyses, as well as the (same) effect size weights of the base GWAS. There were between 569 and 22,513 unrelated individuals for this multiancestry replication.

#### All of Us cohort

The All of Us Research Program is a diverse ancestry biobank from the United States, using electronic health record data in a cohort of individuals with a wider age range than the UKB (mean (s.d.) age of 55.8 (17.1) versus 56.5 (8.1) years). Traits matching those studied in our primary UKB analysis were identified through an initial database search and then interrogated manually to determine whether trait descriptions were sufficiently similar. We identified 31 traits as overlapping with the 74 traits of the primary UKB analysis, available for replication here. All of Us provides ancestry categories that correspond to the labels used in gnomAD^48^ and are based on principal component analysis projection of samples into the principal component analysis space of the training data comprising the Human Genome Diversity Project^49^ and 1000 Genomes project^50^. A total of 133,581 individuals of European ancestry (genetically inferred by All of Us) were extracted for analysis. Trait QC in All of Us was conducted to match that performed in the UKB as closely as possible, given available trait information. First, individuals on statins were removed from the blood count and blood biochemistry subcategories; then, the median measurement value for every trait (electronic health record data includes multiple measures) was selected, and the age corresponding to that measurement was extracted. For each trait, outliers more than six standard deviations from the mean were removed, and a linear regression model was applied with covariates for age, age^2^, age^3^, sex (reported at birth) and the first 40 genetic principal components. A rank inverse normal transformation was applied to the regression residuals to provide standardized trait values, as for the UKB.

Genetic QC in All of Us was conducted using standard filters, as used in the UKB. SNPs with MAF < 0.01, genotype missingness > 0.02, or Hardy–Weinberg equilibrium test *P* < 10^−8^ were excluded. The same greedy algorithm used to maximize sample retention of unrelated individuals in the UKB was applied here. Using the higher-powered publicly available European ancestry GWAS summary statistics generated from the entire UKB^40^ to provide SNP weights, we calculated trait PRSs^47^ using the same *P* value threshold relevant to each trait and trained in the UKB European ancestry analysis.

### POPout analyses

#### The POPout test

The POPout test was designed to identify departures from common-variant architecture in the tails of quantitative traits from population genotype data. In our application, we sought to identify departures due to genetic factors; thus, we first performed regression of traits on environmental risk factors and used the residuals as input to the test. The POPout test first applies a regression of PRS-on-trait values, in which both the PRS and trait are standardized to have a standard deviation of 1. The assumption of the test is that under uniform and predominant common-variant architecture, comprising many common variants of small effect, there is a linear relationship in prediction of PRS from trait values, whereas under reduced common-variant architecture in the tails, the fitted regression values systematically overestimate the absolute PRS values in the lower and upper quantiles (that is, the observed PRS for extreme trait values is closer to the population mean PRS than predicted by linear regression). The residuals from the regression are evaluated in trait quantiles (centiles here), and two-tailed *t*-tests are applied to test whether the residuals are significantly different from zero. Here we applied the test to individuals in the lower and upper 1% of the trait distribution to test for departures from common-variant architecture in the tails. The POPout effect size in each tail was defined such that a positive effect was indicative of regression to the mean in both the lower and upper tails, as follows:

$$\text{POPout effect}\,=\,\frac{1}{n}\mathop{\sum }\limits_{i}^{n}f(\,{y}_{i})-{\mathrm{PRS}}_{i}.(\mathrm{sign}(\,f(\,{y}_{i}))),\,i\in 1,\ldots ,n:{y}_{i} > K,$$

where $K={\varPhi }^{-1}(0.99)$ (testing the top 1% tail), ${\varPhi }^{-1}$ is the inverse normal cumulative density function, $f(\,{y}_{i})$ is the predicted PRS from the regression given trait value ${y}_{i}$ and *n* is the number of observations in the quantile.

#### POPout test QC

A further trait QC was applied to ensure the robustness of the POPout to its assumptions by ensuring that the POPout test did not produce significant results in the middle of the trait distribution. Specifically, for each trait, two-sided *t*-tests for zero mean were applied to the residuals assigned in each of the middle 80 centiles of the trait distribution. Traits were deemed to fail QC if the smallest *P* value was significant at a Bonferroni-corrected level of 0.05 (*P* ≤ 0.05/80). Three traits were removed on the basis of this criterion in our large UKB primary analysis, six of the repeated measures traits, ten traits in the multiancestry replication and three traits in All of Us.

#### Replication of POPout effects

We replicated our primary POPout analyses in three different independent cohorts: (1) UKB European ancestry individuals with repeat measures (baseline and follow-up at approximately 5 years), (2) non-European multiancestry UKB individuals combined into a single group, and (3) the European ancestry samples in the US-based All of Us biobank. Replication POPout analyses were then performed in cohorts (1) and (2) using the PRS models trained in the primary analysis (that is, the same variants and effect size weights for the PRS), and likewise for cohort (3) except that the effect size weights of the variants were derived from the total UKB sample of individuals with European ancestry, as the All of Us sample is entirely independent and thus cannot be subject to overfitting.

In our primary analysis, we set a minimum total sample size threshold of 100,000 (at least 50,000 target samples). To set an appropriate minimum sample size for replication, we explored the power of POPout at different sample sizes, applying POPout to subsets ranging from 1,000 to 50,000 target samples, with subset sizes incremented by 5,000 (Extended Data Fig. 3). Analyses were repeated 100 times for each sample size. Results were compared with the primary analysis with respect to (1) the number of false discovery rate 5% significant tails replicated at *P* < 0.05 and (2) the average (Pearson) correlation across POPout effects between the primary and subset analyses. As shown in Extended Data Fig. 3, at subset sizes of 5,000, the mean correlation across bootstraps was 0.72, whereas at a sample size of 1,000 the mean correlation was 0.41. A threshold of 5,000 was therefore used as a minimum for each cohort in the replication analyses.

After performing the POPout test QC and applying a sample size threshold of >5,000 individuals, we had 50 traits (of 63 overlapping traits) for the repeated measures replication across an average of approximately 12,000 individuals, 55 traits (of 74 traits) for the multiancestry replication across an average of approximately 17,500 individuals, and 27 (of 31 overlapping traits) for the All of Us replication across an average of approximately 53,000 individuals.

#### Evaluating impact of POPout effects on PRS accuracy

To evaluate how observed POPout effects affected the performance of PRS prediction, we emulated a scenario in which the quantitative trait underlies a binary outcome (for example, body mass index underlying clinical obesity). We considered individuals above a specified trait threshold value to be cases and individuals below the threshold to be controls. We investigated thresholds at the 75% and 99% points of the trait distribution. For each, we used the PRS to predict case or control status and then generated a quantile plot in which the odds ratio of the given quantile was estimated in relation to the central quantile by means of logistic regression (Fig. 2c). In addition, for each trait with PRS *r*^2^, we simulated a quantitative phenotype with trait values given by $T={r}{\rm{P}}{\rm{R}}{\rm{S}}+E$, where PRS has a standard normal distribution and $E$ represents all other contributions to trait variance and is drawn from a normal distribution with mean 0 and variance $(1-{r}^{2})$. This produced a simulated trait with ${\mathcal{N}}(0,1)$ distribution and PRS *r*^2^. Using the same number of target samples as in the real data analysis for each trait, this simulation allowed us to estimate the expected predictive performance of the PRS on the basis of the corresponding *r*^2^, assuming uniform polygenicity throughout the trait distribution. The results of this analysis for all our index traits are shown in Extended Data Fig. 2.

### Sibling-based analyses

#### Sibling-based investigation of departures from common-variant architecture

We previously developed sibling-based tests of departures from polygenic expectations in trait tails caused by de novo high-impact alleles or rare alleles of large effect segregating in families (Mendelian effects)^2^. Here we summarize the theoretical framework underlying those tests and describe the single omnibus test used here that combines the two tests to identify general departures from common-variant architecture due to either cause.

The conditional distribution of an individual’s trait value given their sibling’s trait value, assuming the infinitesimal model for genetic effects (common-variant architecture) and normally distributed environmental factors, can be derived analytically^2^ as

$$p({s}_{2}|{s}_{1}) \sim {\mathcal{N}}\,\left(\frac{1}{2}{s}_{1}{h}^{2},1-\frac{{h}^{4}}{4}\right),$$

where ${s}_{1}$ and ${s}_{2}$ denote the index and conditional-sibling trait values, respectively, and ${h}^{2}$ is the trait heritability. Rare large-effect alleles can produce deviations from this distribution, particularly when concentrated in specific parts of the distribution, as may occur in the trait tails if these effects are very large and abundant. Specifically, rare large-effect alleles segregating in families (Mendelian effects) will result in excess trait similarity (or concordance) between siblings on average compared with a common-variant only architecture. Conversely, a high-impact de novo mutation in one sibling will result in excess dissimilarity (discordance) between siblings.

Our sibling-based test for enrichment of Mendelian effects^2^ evaluates the proportion of sibling pairs in which both siblings reside in a defined quantile of the trait distribution; this can be formulated as a score test with the following *z*-statistic:

$$z=\frac{r-n{\pi }_{0}}{\sqrt{n{\pi }_{0}(1-{\pi }_{0})}},$$

where $n$ is the number of index siblings in quantile $q$ under test, $r$ is the number of sibling pairs in which both siblings have trait values in $q$. ${\pi }_{0}$ is the expected proportion of sibling pairs in the quantile under common-variant architecture, given by:

$${\pi }_{0}=\frac{1}{n}\sum _{i\in {A}_{q}}\left(1-\varPhi \left(\frac{{\varPhi }^{-1}(q)-\frac{{s}_{1}^{(i)}{h}^{2}}{2}}{\sqrt{1-\frac{{h}^{4}}{4}}}\right)\right),$$

where *n* is the number of sibling pairs, ${A}_{q}$ is the set of index siblings in trait quantile *q*, $\varPhi$ is the normal cumulative density function and ${\varPhi }^{-1}$ is its inverse.

Our sibling-based test for enrichment of de novo effects constructs a *z*-statistic for the distance between index siblings in the tail of the trait distribution and their conditional siblings, as follows:

$$z=\frac{\sum _{i\in {A}_{q}}{s}_{{2}^{(i)}}-\frac{1}{2}{s}_{1}^{(i)}{h}^{2}}{\sqrt{n\left(1-\frac{{h}^{4}}{4}\right)}}.$$

For index siblings in the lower and upper tails, the alternative hypotheses of ${H}_{1}:z > 0$ and ${H}_{1}:z < 0$ are tested as one-tailed *t*-tests, respectively. For further details, see Souaiaia et al.^2^.

The trait heritability, ${h}^{2}$, a required parameter in the Mendelian and de novo tests, is estimated from the sibling pairs by calculating the maximum likelihood of ${h}^{2}$ under the null hypothesis of the infinitesimal model:

$$\log {\mathcal{L}}\propto -n\log \left(1-\frac{{h}^{4}}{4}\right)-\frac{1}{1-\frac{{h}^{4}}{4}}\mathop{\sum }\limits_{i=1}^{n}{\left({s}_{2}^{(i)}-\frac{{s}_{1}^{(i)}{h}^{2}}{2}\right)}^{2}.$$

#### The sibling STANDout test

To test for general departures from common-variant architecture in the tails of quantitative traits, caused by high-effect alleles that are either de novo or segregating in families (that is, rare alleles), we combine the results of the two tests using Fisher’s method to give a ${\chi }^{2}$ statistic with four degrees of freedom:

$${\chi }_{4}^{2}=-2({\rm{l}}{\rm{o}}{\rm{g}}({P}_{{\rm{M}}{\rm{e}}{\rm{n}}{\rm{d}}{\rm{e}}{\rm{l}}{\rm{i}}{\rm{a}}{\rm{n}}})+{\rm{l}}{\rm{o}}{\rm{g}}({P}_{{\rm{denovo}}})).$$

### Analysis of rare variants

#### Calculating associations between POPout effects and rare exome hits

Backman et al.^26^ provided association results from the UKB exome sequencing study across 3,994 traits. These results included 65 of the 74 traits analysed in our target set. For each of these 65 traits, in each tail, we counted the number of genes with at least one significant association. Following Backman et al.^26^, we defined gene significance as either a single-variant deleterious, missense or predicted loss-of-function association, or a gene-burden association, at *P* $\le \,2.18\times {10}^{-11}$. Across the 130 trait tails, we calculated the Pearson correlation between the number of genes with significant exome associations and the POPout effect size.

#### WGS data to identify rare variants

The UKB recently released WGS data for approximately 500,000 participants^27^. This dataset has an average coverage depth of 23.5×, enabling reliable detection of rare genetic variants (MAF < 1%) typically absent from PRS analyses. We analysed the European-ancestry samples that had previously undergone QC using the same standardized 74 traits. GWAS (with standard QC filters, variant missingness < 0.02), followed by clumping and thresholding (${r}^{2}=0.1$, window: 250 kb) were applied using PLINK-1.9 to identify independent rare associations^43,44^ with 0.01% < MAF<1% in the full dataset. In addition to the covariates used in the common-variant GWAS, the top 40 rare-variant principal components were included as covariates in the GWAS, after which independent variants meeting the criteria for genome-wide significance ($P < 2.18\times {10}^{-11}$), as described by Backman et al.^26^, were retained and separated into two groups on the basis of MAF. Variants in each group were summed across sample genotypes to build moderately rare (0.1% < MAF < 1%) and very rare (0.01% < MAF < 0.1%) PRS models for each trait.

#### WES data to identify rare burden variants

The UKB has released WES data for over 450,000 of 500,000 participants^26^, enabling gene-level burden variants to be tested for association with quantitative traits. For the 40 traits with at least one genome-wide significant rare-variant and significant positive POPout, we used interim-release PLINK-format WES data that had undergone QC to further identify burden variants. First, we applied REGENIE v.4.1 (ref. ^51^) to fit chromosome-level whole-exome REGENIE null models (REGENIE step 1) using all available samples (passing our original QC) and a pruned set of high-quality variants (MAF > 1%, LD pruning window: 50 kb, ${r}^{2} < 0.2$) to generate leave-one-chromosome-out predictions that controlled for population structure and relatedness.

Next, we performed gene-based burden testing (REGENIE step 2) in the base sample subset (in which GWAS were performed in the primary analyses) using leave-one-chromosome-out predictors from step 1 and the standard four functional masks defined in the UKB exome files, capturing rare (MAF < 1%) and singleton loss-of-function and damaging missense variants. Burden effect sizes ($\beta$) and corresponding *P* values were obtained for each gene–mask pair. To avoid double-counting of rare variants within traits, we excluded gene–mask pairs containing variants overlapping with our single-variant rare PRS analysis, which will have reduced the specific power of our burden-based analyses (but not the power of the overall rare-variant analyses). Next, we selected the most significant mask per gene, consistent with standard burden-based frameworks^26,52^, and retained those with *P* values exceeding genome-wide significance ($P < 2.18\times {10}^{-11}$)^26^. Per-sample burden genotype matrices for individuals in the target sample (the second half of the UKB sample used for POPout analyses) were then multiplied by the corresponding burden $\beta$ coefficients and summed across chromosomes to compute burden-variant PRS in the target sample for each trait.

#### Construction and analysis of common + rare PRS

For each trait, the union of target samples from our original common-variant PRS and samples available in the WGS and WES datasets (more than 99% of sample shared) were used to calculate a combined common + rare PRS that was summed across common risk scores (${MAF} > 1 \%$), moderately rare single-variant risk scores ($0.1 \%  < {MAF} < 1 \%$), very rare single-variant risk scores ($0.01 \%  < {MAF} < 0.1 \%$) and burden-variant risk scores. For the 40 traits (of 74 traits in total) with significant rare variants identified and positive POPout effects, common-variant PRSs were used to recalculate POPout effects in the overlapping samples (with genotype, WES and WGS data), and common + rare-variant PRSs were used to calculate new POPout effects that incorporated rare variants. To compare these POPout values, we quantified the ‘POPout reduction’ as follows:

$$\mathrm{POPout}\,\mathrm{reduction}\,=\,\frac{{{\rm{POPout}}}_{{\rm{common}}}-{\rm{POPou}}{{\rm{t}}}_{{\rm{common}}+{\rm{rare}}}}{{\rm{POPou}}{{\rm{t}}}_{{\rm{common}}}}$$

and evaluated whether the reduction was statistically significant using a one-sided paired *t*-test on the within-individual differences between POPout effects.

In the odds ratio analyses, odds ratios were calculated for PRS prediction of a hypothetical disease defined as corresponding to the lower or upper 1% tail, for each trait tail before and after inclusion of rare variants (that is, corresponding to odds ratios calculated using only the common PRS versus odds ratios calculated using the common + rare PRS).

### Selection analyses

#### Forward-in-time population genetic simulations

Using SLiM v.4.0 (ref. ^17^), we simulated a quantitative polygenic trait under a standard Wright–Fisher model of stabilizing selection. First, a population of *N* = 10,000 diploid individuals of genome size 100 kb was simulated to equilibrium (*N* generations; as well as sensitivity analyses of 5*N* and 10*N* generations) with a constant mutation rate of $2.36\times {10}^{-8}$ per base pair and recombination rate of ${10}^{-8}$ per base pair under neutrality. Mutations were assigned effect sizes simulated from a gamma distribution with shape parameter 0.186 and mean of 0.01026 and subsequently multiplied by −1 with probability 0.5 to give a symmetrical effect size distribution. This distribution has been used previously to simulate genetic effects in humans and reflects a prior belief in a small number of large effects and many small effects^53^. In sensitivity analyses, gamma distributions with means of 0.005, 0.01 and 0.02 (shape 0.186), as well the standard Gaussian distribution, were used instead for the effect sizes. Individual trait values were determined by the sum of all genetic effects, corresponding to full heritability (${h}^{2}=1$).

After neutral genetic diversity had been achieved (10*N* generations), stabilizing selection on the trait was introduced for *N* (5*N*, 10*N* in sensitivity analyses) generations through a Gaussian fitness function

$${f}_{t}(x)=1+\frac{k}{\sqrt{2\pi {\sigma }^{2}}}\exp \left\{-\frac{{(x-\mu )}^{2}}{2{\sigma }^{2}}\right\},$$

where *k* is a multiplier for strength of selection, *x* is the phenotype of an individual at generation *t*, and $\mu$ and $\sigma$ are the trait mean and standard deviation at the start of selection (that is, $t=10N$).

We ran this simulation for 100 independent replicates and reported data in each simulation by logging, at each generation, the phenotypic mean and standard deviation of the population, as well as statistics related to the mutations present in the populations, including allele frequencies and effect sizes, and the mutational profile of each individual. To analyse the enrichment of rare-variant architecture at different quantiles, the population was stratified across 100 centiles (each including 100 individuals), and the number of rare and common alleles carried by individuals in each centile was calculated under neutrality versus under selection. For comparison purposes, ‘weak selection’ corresponded to simulations performed using a multiplier of $k=1$ in the fitness function equation above, whereas ‘strong selection’ corresponded to simulations with $k=100$. ‘Large effects’ were considered to be those in the top 10% of effect sizes genome-wide. The mean of the log-transformed odds ratio across all 100 independent simulation replicates was used as a measure of enrichment, and a *t*-test was performed to test for significant differences from zero. To analyse the impact of low-frequency variants on prediction and mimic the calculation of common-variant PRS in real data, a PRS was calculated using only variants with MAF > 1% and compared with the true trait value across the individuals in each centile. The mean difference (POPout effect) and 95% confidence intervals in each centile were calculated across the 100 independent simulation replicates. These simulations reflect a subset of those performed in a recent simulation study that we conducted^54^.

#### Inferring selection on the basis of relative lifetime reproductive success

The number of offspring is recorded in the UKB for both females and males. Following the approach of Beauchamp^55^, samples were stratified into four birth cohorts (1934–1942, 1943–1948, 1949–1955, 1956–1965). Individual relative lifetime reproductive success, ${Y}_{{\rm{rLRS}}}$, was then calculated by dividing the number of offspring of each individual by the cohort mean. Following Sanjak et al.^16^, we inferred the presence and direction of selective pressure acting on traits by fitting the following regression model:

$${Y}_{{\rm{rLRS}}}={\beta }_{0}+\beta z+\gamma {z}^{2},$$

where *z* denotes the residualized and normalized trait values, and $\beta$ and $\gamma$ are linear and quadratic regression parameters.

We used the BIC to select the best-fitting model for each trait, from which the selective pressure acting on each trait was inferred. If the selected model did not include a linear or quadratic parameter, then no selection was inferred. If the selected model included only the linear term, then negative selection was inferred if $\beta  < 0$, whereas positive selection was inferred if $\beta  > 0$. If the selected model did not include a linear term but included the quadratic term, then stabilizing selection was inferred if $\gamma  < 0$, suggesting that the mean trait value in the population was optimal; if instead $\gamma  > 0$, it was considered that both tails of the distribution were subject to positive selection (this is known as ‘disruptive selection’ and is expected to be rare in practice). When the BIC-selected model included both linear and quadratic terms, the $\beta$ parameter was used to indicate the general direction of selection as positive or negative, and the quadratic term was considered to suggest that an optimal mean was being reached.

#### Association between POPout effects and lifetime reproductive success

To test the association between POPout effects and the selection acting on corresponding traits as inferred by the lifetime reproductive success model, we stratified traits into four categories of selection. The first two of the four categories reflected non-directional selection: (1) no selection ($\beta =0,\gamma =0$), in which POPout effects were not expected in either tail; and (2) non-directional stabilizing selection ($\beta =0,\gamma  < 0$), in which we expected POPout effects in both tails of equal magnitude. The third and fourth categories reflected directional selection: (3) positive selection ($\beta  > 0$), which could be linear ($\gamma =0$), disruptive ($\gamma  > 0$) or stabilizing ($\gamma  < 0$), in which we expected greater POPout effects in the lower tail versus the upper tail; and (4) negative selection ($\beta  < 0$; linear, disruptive or stabilizing as for positive), in which we expected greater POPout effects in the upper tail versus the lower tail. For each category, a *t*-test was used to test for the difference in mean POPout effects (excluding non-significant POPout effects) between the lower and upper tails across the traits.

#### Sensitivity of lifetime reproductive success analyses to BIC model choice

For each pair of best and second-best models, as ranked by BIC, we performed the following comparison. When the second-best model was a nested subset of the best model, we calculated the likelihood ratio test to determine whether it was significantly better-fitting (*P* < 0.05). When the second-best model had more parameters, we determined that a difference in BIC of less than 2 was weak evidence of model superiority^56^. These two criteria identified ten and seven traits, respectively, for which there was justification to test the second-best model. These traits are shown in the Supplementary Information. In sensitivity analyses, we also replaced the best model with the second-best model for these 17 traits and then re-estimated the relationship between LRS and POPout effects observed in Fig. 5d (Supplementary Information).

#### Calculating association between POPout effects and fitness measures

Five broad measures of fitness were considered: (1) male and (2) female fecundity (field IDs: 2405 and 2734), (3) self-reported non-cancer illnesses (field ID: 2002), (4) total miscarriages and stillbirths (field IDs: 39290 and 3839), and (5) paternal age (calculated as the difference between father’s age (field ID: 2946) and participant age (field ID: 21022)). The association between each of these measures and the POPout effects was calculated across the lower and upper (1%) tails, as in the following example. To test for the association between paternal age and POPout effects, the mean paternal age of the individuals residing in the upper tail and lower tail was calculated for each of the 74 traits, resulting in a set of 148 mean paternal age values. These 148 values were then tested for their associations with the corresponding 148 POPout effect sizes (two POPout effects, one for each of the lower and upper tails, across the 74 traits) using Spearman’s rank correlation. All analyses and QC described here can be repeated, modified or expanded using our publicly available scripts^57^.

### Reporting summary

Further information on research design is available in the Nature Portfolio Reporting Summary linked to this article.

## Online content

Any methods, additional references, Nature Portfolio reporting summaries, source data, extended data, supplementary information, acknowledgements, peer review information; details of author contributions and competing interests; and statements of data and code availability are available at 10.1038/s41586-026-10516-5.

## Supplementary information

Supplementary Information Tables and figures of results corresponding to sensitivity analyses performed in relation to the lifetime reproductive success modelling and testing. Supplementary Table 1 shows results from replication of work by Sanjak et al.^16^ and Fig. 1 shows results of sensitivity analyses related to optimization of the BIC model.

Reporting Summary

Supplementary Data Source data for Supplementary Fig. 1.

Peer Review File

## Source data

Source Data Fig. 2

Source Data Fig. 3

Source Data Fig. 4

Source Data Fig. 5

Source Data Extended Data Fig. 1

Source Data Extended Data Fig. 2

Source Data Extended Data Fig. 3

Source Data Extended Data Fig. 4

Source Data Extended Data Fig. 5

Source Data Extended Data Fig. 6

Source Data Extended Data Fig. 7

## Figures and Tables

**Extended Data Table 1:**
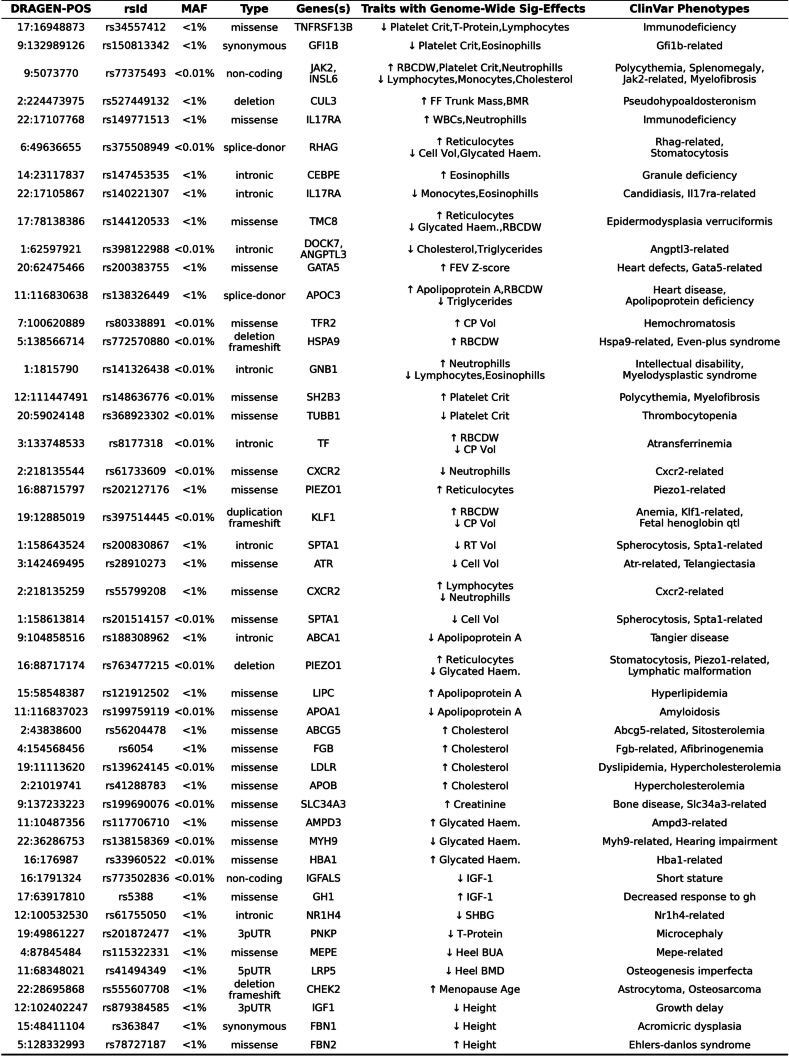
Rare variants identified in the whole-genome sequence analysis that are present in ClinVar database

Annotation table comprising rare variants identified as significant in whole-genome sequence analyses, which are then incorporated into our “rare PRS”, that also appear in the ClinVar database^28^.

**Extended Data Table 2:**
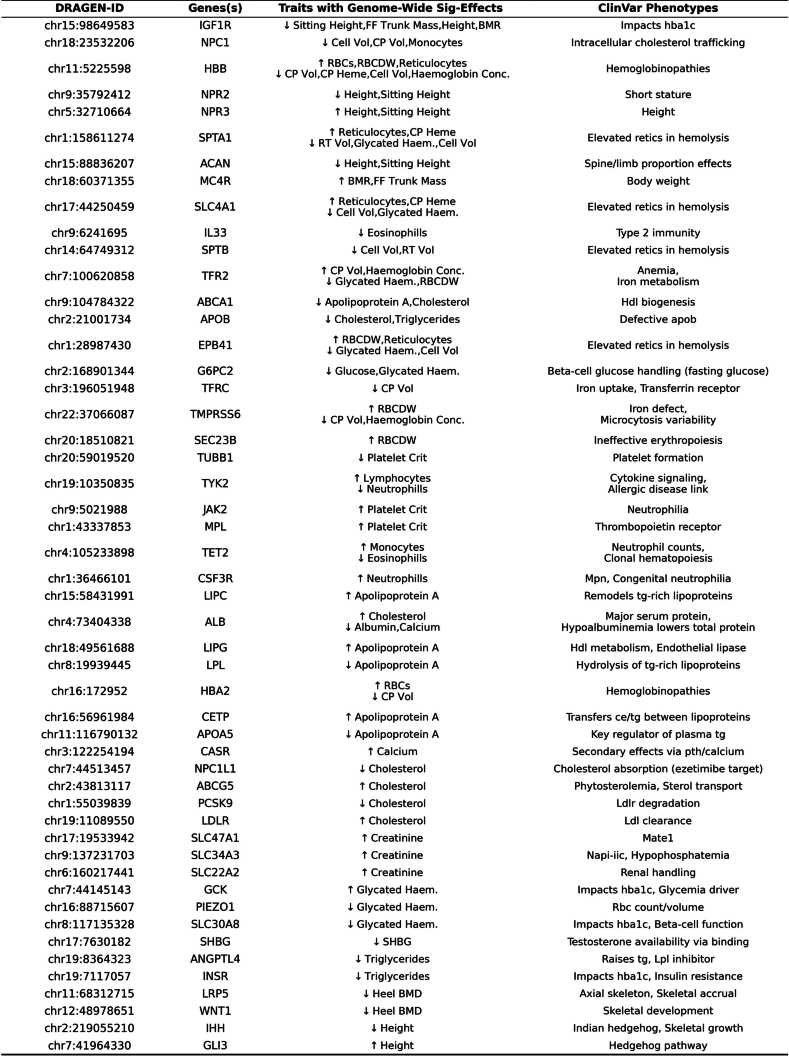
Rare variants identified in the rare burden analysis that are present in the ClinVar database

Annotation table comprising rare variants identified as significant in rare burden analyses, which are then incorporated into our “rare PRS”, that also appear in the ClinVar database^28^.

**Extended Data Table 3:**
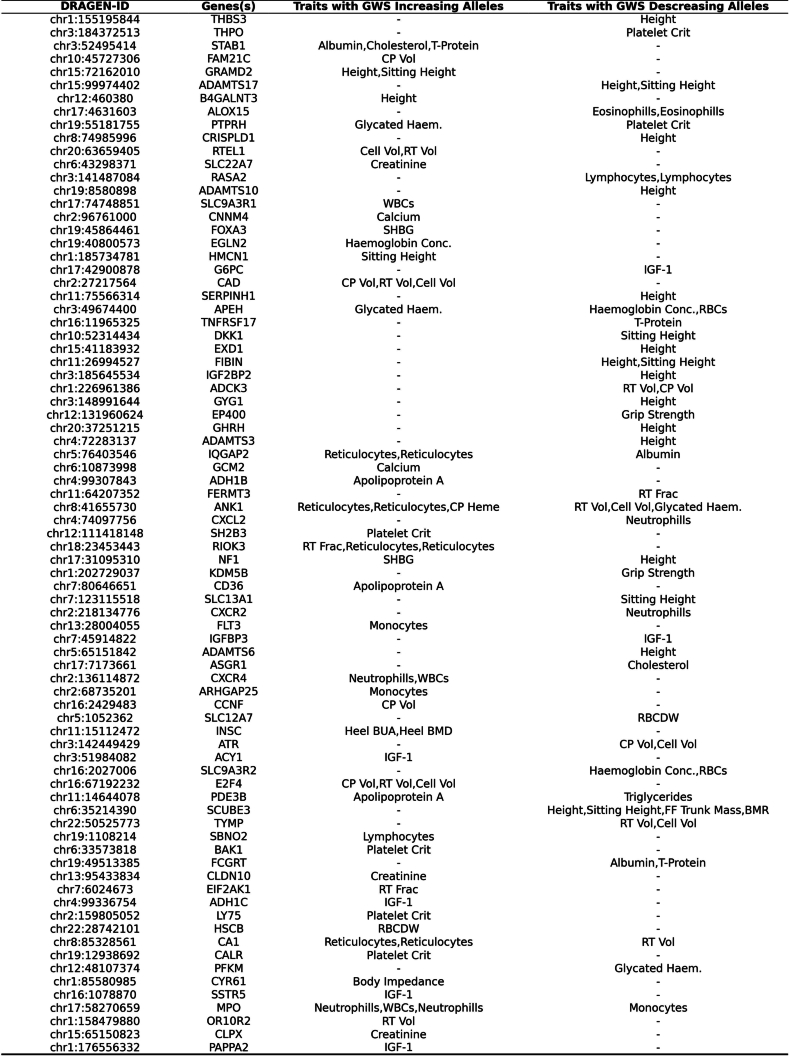
Rare variants identified in the rare burden analysis that are not present in the ClinVar database

Annotation table comprising rare variants identified as significant in rare burden analyses, which are then incorporated into our “rare PRS”, that do not appear in the ClinVar database^28^.

## Data Availability

UKB genotype and phenotype data were obtained from the UKB Resource under application 18177 to P.F.O. General information can be found at: www.ukbiobank.ac.uk/enable-your-research/approved-research/multi-trait-gwas-analyses-in-the-uk-biobank. UKB QC information (missingness, allele frequency, Hardy–Weinberg equilibrium) was obtained from UKB resource 531: www.biobank.ctsu.ox.ac.uk/crystal/refer.cgi?id=531. All of Us data are available through application to the All of Us Research Program workbench. GWAS summary statistics are available from: www.nealelab.is/uk-biobank. UKB exome sequencing study data and results are available from: www.app.genebass.org. Source data are provided with this paper.

## Code availability

Scripts for performing all analyses and QC and generating all figures and tables presented in the manuscript are available at Zenodo (10.5281/zenodo.20195343)^57^. POPout and STANDout software tools are written in Python and R and are available on dedicated websites (www.tailstests.org and www.sibarc.net). These include download links to example data, required packages, user options, input file formatting and output files, and a quickstart guide. These and other code used in this study are available from the following websites: LDSC (v.1.0.1), https://github.com/bulik/ldsc; METAL (v.2011-03-25), http://csg.sph.umich.edu/abecasis/metal/; PLINK v.1.9, https://www.cog-genomics.org/plink; PLINK v.2.0, https://www.cog-genomics.org/plink/2.0/; POPout v.0.0.3, www.tailstests.org; PRSice-2, https://www.prsice.info; R (v.4.0.3), https://cran.r-project.org; R package boot (v.1.3.25), https://cran.r-project.org/web/packages/boot/index.html; STANDout v.1.0.2, www.sibarc.net; SLiM v.4.0, https://messerlab.org/slim/.
