# Supplementary material for: Distinct genetic architecture in the tails of complex traits
Source: Nature. 2026 May 27;655(8123):676–84. doi: 10.1038/s41586-026-10516-5 (PMC13372694; doi:10.1038/s41586-026-10516-5)
Supplement: Supplementary file 1 — Tables and figures of results corresponding to sensitivity analyses performed in relation to the lifetime reproductive success modelling and testing. Supplementary Table 1 shows results from replication of work by Sanjak et al.16 and Fig. 1 shows results of sensitivity analyses related to optimization of the BIC model. [file 41586_2026_10516_MOESM1_ESM.pdf]

nature portfolio

https://doi.org/10.1038/s41586-026-10516-5

Supplementary information

Distinct genetic architecture in the tails of complex traits

In the format provided by the authors and unedited

Supplementary Information

Trait Name [UKB ID]	Model Selection			Sanjak et al					
	Top Model	Linear Parameter (β)	Quadratic Parameter (γ)	Aggregate Result		Males		Females	
				β	γ	β	γ	β	γ
Traits Without Sex Differences									
Body Mass Index (bmi) (21001)	$Y \sim X_o + X + X^2$	0.034 (positive)	-0.025 (stabilising)	positive	stabilising	0.072*	-0.0327*	0.0601*	-0.022*
Basal Metabolic Rate (23105)	$Y \sim X_o + X + X^2$	0.042 (positive)	-0.023 (stabilising)	positive	stabilising	0.0747*	-0.03*	0.0405*	-0.019
Birth Weight (20022)	$Y \sim X_o + X$	0.011 (positive)	0 (neutral)	positive	neutral	0.0208*	-0.0016	0.0047	-0.005
Body Fat Percentage (23099)	$Y \sim X_o + X^2$	0 (neutral)	-0.025 (stabilising)	positive	stabilising	0.0198*	-0.0273*	0.0188*	-0.016*
FEV Best (20150)	$Y \sim X_o + X + X^2$	0.023 (positive)	-0.011 (stabilising)	positive	stabilising	0.0335*	-0.0109*	0.0126	-0.004*
FVC Best (20151)	$Y \sim X_o + X + X^2$	0.026 (positive)	-0.013 (stabilising)	positive	stabilising	0.0323*	-0.0141*	0.0165*	-0.007*
Match Time (20023)	$Y \sim X_o + X + X^2$	-0.026 (negative)	-0.007 (stabilising)	negative	stabilising	-0.0434*	-0.01*	-0.0101*	-0.0002
Pulse Rate (4194)	$Y \sim X_o + X + X^2$	-0.031 (negative)	-0.011 (stabilising)	negative	stabilising	-0.0316*	-0.0062*	-0.0136*	-0.0053*
Waist Circumference (48)	$Y \sim X_o + X + X^2$	0.032 (positive)	-0.027 (stabilising)	positive	stabilising	0.0459*	-0.0331*	0.0649*	-0.028*
Weight (21002)	$Y \sim X_o + X + X^2$	0.035 (positive)	-0.028 (stabilising)	positive	stabilising	0.0761*	-0.0343*	0.0439*	-0.0213*
Traits With Sex Differences									
Edu Age (845)	$Y \sim X_o + X + X^2$	-0.044 (negative)	0.003 (disruptive)	positive, negative	disruptive	0.0086	0.0313*	-0.0612*	0.034*
Fluid Intelligence Score (20016)	$Y \sim X_o$	0 (neutral)	0 (neutral)	neutral	neutral	0.0139	0.0009	-0.0473	0.02
Hand Grip Strength (left) (46)	$Y \sim X_o + X$	0.045 (positive)	0 (neutral)	positive	neutral	0.0728*	-0.0023	0.0124*	0.003
Heel Bone Mineral Density (3148)	$Y \sim X_o + X^2$	0 (neutral)	-0.014 (stabilising)	positive, negative	stabilising	0.0325*	-0.0094*	-0.0105	-0.01*
Standing Height (50)	$Y \sim X_o + X^2$	0 (neutral)	-0.019 (stabilising)	positive, negative	stabilising	0.0229*	-0.0152*	-0.0283*	-0.0188*
Neuroticism Score (20127)	$Y \sim X_o + X + X^2$	-0.02 (negative)	-0.011 (stabilising)	negative, positive	stabilising	-0.0318*	-0.006	-0.0003	-0.0061*

Supplementary Table 1: Replication of Sanjak et al. [16] lifetime reproductive success results. Comparison of results among overlapping traits (without sex differences) shows replication (i.e. statistically significant parameters included in our model) for 19 of 20 parameters. The only exception is *Body Fat*, in which Sanjak et al [16] reported a positive stabilising result in males and females, while we selected a neutral stabilising model in the pooled sample.

a

Nested Alternatives

Trait	Top Model			Alternate Model	
	Model	Selective Pressure	Model Confidence (LRT P-Value)	Model	Selective Pressure
Albumin	$Y \sim X_o - X^2$	stabilising	>0.1	$Y\ X_o$	neutral
Alphanum. Trail	$Y \sim X_o - X$	negative	>0.05	$Y\ X_o$	neutral
Calcium	$Y \sim X_o - X^2$	stabilising	>0.05	$Y\ X_o$	neutral
Cholesterol	$Y \sim X_o - X^2$	stabilising	>0.1	$Y\ X_o$	neutral
Haematocrit %	$Y \sim X_o + X$	positive	>0.1	$Y\ X_o$	neutral
IGF-1	$Y \sim X_o - X - X^2$	neg,stabilising	>0.05	$Y\ X_o - X^2$	stabilising
Monocyte %	$Y \sim X_o - X^2$	stabilising	>0.1	$Y\ X_o$	neutral
Sphered Cell Vol	$Y \sim X_o - X - X^2$	neg,stabilising	>0.1	$Y\ X_o - X$	negative
Testosterone	$Y \sim X_o - X - X^2$	neg,stabilising	>0.05	$Y\ X_o - X^2$	stabilising
WBCs	$Y \sim X_o - X - X^2$	neg,stabilising	>0.1	$Y\ X_o - X$	negative

Complex Alternatives

Trait	Top Model			Alternate Model	
	Model	Selective Pressure	ΔBIC	Model	Selective Pressure
Birth Weight	$Y \sim X_o + X$	positive	0.4	$Y\ X_o + X - X^2$	pos,stabilising
Body Fat %	$Y \sim X_o - X^2$	stabilising	0.2	$Y\ X_o + X - X^2$	pos,stabilising
Mean CP Vol	$Y \sim X_o - X$	negative	1.3	$Y\ X_o - X - X^2$	neg,stabilising
Numeric Trail	$Y \sim X_o$	neutral	1.1	$Y\ X_o + X$	positive
Phosphate	$Y \sim X_o$	neutral	0.3	$Y\ X_o + X$	positive
Platelet Crit	$Y \sim X_o - X$	negative	0.6	$Y\ X_o - X - X^2$	neg,stabilising
Sodium In Urine	$Y \sim X_o - X^2$	stabilising	0.4	$Y\ X_o + X - X^2$	pos,stabilising

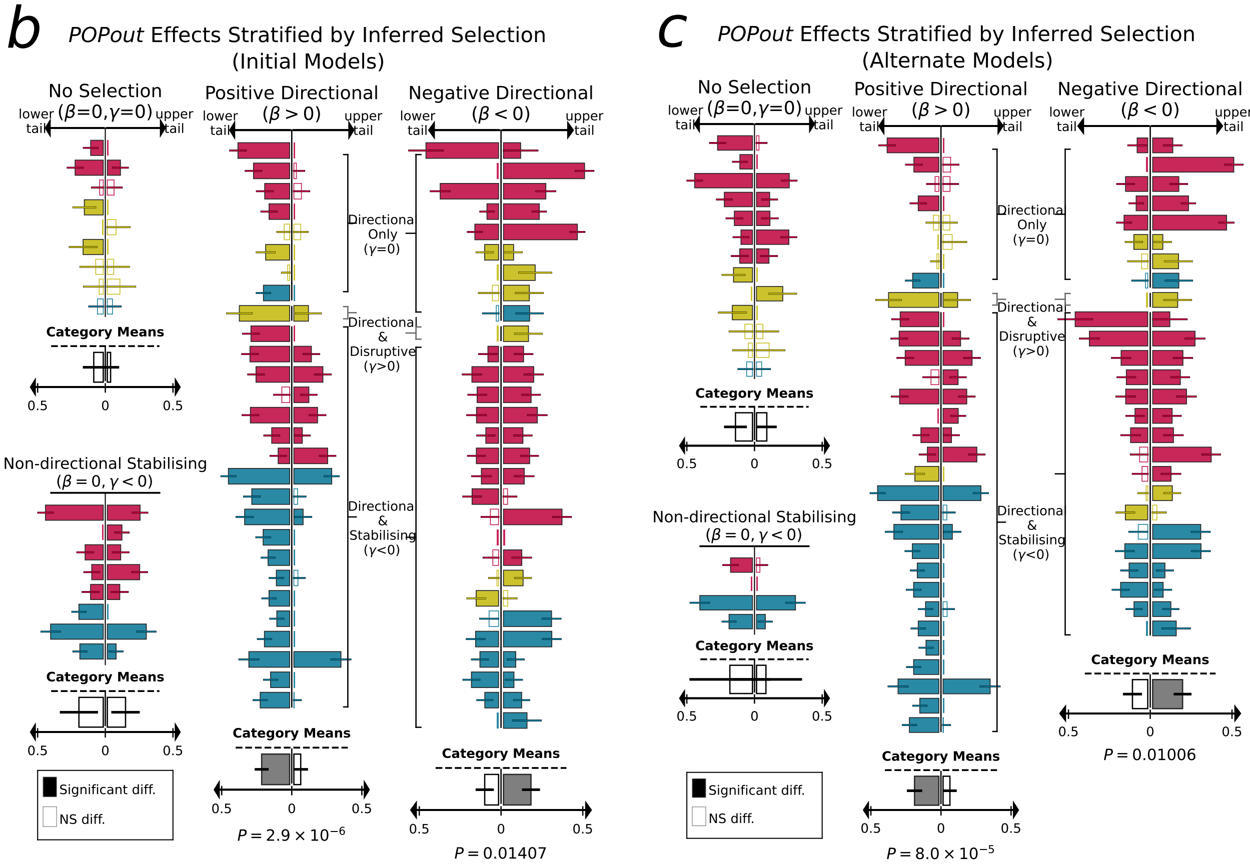

Supplementary Figure 1: BIC model selection sensitivity analysis. a, For 17 of 74 traits, the top model selected using BIC was not significantly better fitting than the second best model. **b,** Association between BIC selected models of selection and POPout effects (top models). These present the same results as in Figure 5d and are shown here only for convenience in visual comparison with the results of panel c in which alternative models are tested. **c,** Association between BIC selected models of selection and POPout effects after instead using the second best model for the 17 traits in which the top model was not significantly better fitting than the second best model. As in Figure 5d, POPout effect sizes (with 95% CIs) for traits are grouped by selection category inferred by BIC model (models with linear and quadratic terms are categorised by direction of linear term). Differences between lower and upper tail effects tested by two-sided t-test in each of the four categories. Note that negative POPout effects are shown as having POPout effects of 0 (without CIs shown) for illustrative convenience; testing was performed on the actual POPout effects.
