# Supplementary material for: Distinct genetic architecture in the tails of complex traits
Source: Nature. 2026 May 27;655(8123):676–84. doi: 10.1038/s41586-026-10516-5 (PMC13372694; doi:10.1038/s41586-026-10516-5)
Supplement: Supplementary file 2 — Reporting Summary [file 41586_2026_10516_MOESM2_ESM.pdf]

nature portfolio

Corresponding author(s): Tade Souaiaia
Hei Man (Beatrice) Wu
Paul F. O'Reilly

Last updated by author(s): Jan 28, 2026

Reporting Summary

Nature Portfolio wishes to improve the reproducibility of the work that we publish. This form provides structure for consistency and transparency in reporting. For further information on Nature Portfolio policies, see our Editorial Policies and the Editorial Policy Checklist.

Statistics

For all statistical analyses, confirm that the following items are present in the figure legend, table legend, main text, or Methods section.

n/a	Confirmed	
☐	☒	The exact sample size (*n*) for each experimental group/condition, given as a discrete number and unit of measurement
☐	☒	A statement on whether measurements were taken from distinct samples or whether the same sample was measured repeatedly
☐	☒	The statistical test(s) used AND whether they are one- or two-sided<br>*Only common tests should be described solely by name; describe more complex techniques in the Methods section.*
☐	☒	A description of all covariates tested
☐	☒	A description of any assumptions or corrections, such as tests of normality and adjustment for multiple comparisons
☐	☒	A full description of the statistical parameters including central tendency (e.g. means) or other basic estimates (e.g. regression coefficient) AND variation (e.g. standard deviation) or associated estimates of uncertainty (e.g. confidence intervals)
☐	☒	For null hypothesis testing, the test statistic (e.g. *F*, *t*, *r*) with confidence intervals, effect sizes, degrees of freedom and *P* value noted<br>*Give P values as exact values whenever suitable.*
☒	☐	For Bayesian analysis, information on the choice of priors and Markov chain Monte Carlo settings
☐	☒	For hierarchical and complex designs, identification of the appropriate level for tests and full reporting of outcomes
☐	☒	Estimates of effect sizes (e.g. Cohen's *d*, Pearson's *r*), indicating how they were calculated

Our web collection on statistics for biologists contains articles on many of the points above.

Software and code

Policy information about availability of computer code

Data collection	No software was used by the authors to collect the data in this study.
Data analysis	The POPout and STANDout tests and analytical pipelines were coded by the authors in python3 and R. Both software packages are publicly and freely available to users on dedicated websites that the authors maintain: at www.tailstests.org and www.sibarc.net, respectively. These websites include download links to sample data, installation and users instructions, and quickstart tutorials so that users can run these tests on any available POP (Phenotype-on-PRS) or Sib (paired sibling phenotype) input data, as described on the websites.<br>All code used in this study is available from the following websites<br>- PLINK v1.90: https://www.cog-genomics.org/plink/1.9/<br>- PLINK v2: https://www.cog-genomics.org/plink/2.0/<br>- PRSice-2 (https://choishingwan.github.io/PRSice/}<br>- POPout v0.0.3 (www.tailstests.org)<br>- STANDout v1.0.2 (www.sibArc.net)<br>- LDSC version v1.01: https://github.com/bulik/ldsc<br>- SLiM v4.0 (https://messerlab.org/slim/)<br>- REGENIE v4.1 (https://rgcgithub.github.io/regenie/)<br>- R version 4.0.3 https://cran.r-project.org

For manuscripts utilizing custom algorithms or software that are central to the research but not yet described in published literature, software must be made available to editors and reviewers. We strongly encourage code deposition in a community repository (e.g. GitHub). See the Nature Portfolio guidelines for submitting code & software for further information.

Data

Policy information about availability of data

All manuscripts must include a data availability statement. This statement should provide the following information, where applicable:

- Accession codes, unique identifiers, or web links for publicly available datasets
- A description of any restrictions on data availability
- For clinical datasets or third party data, please ensure that the statement adheres to our policy

UK Biobank genotype and phenotype data were obtained under approved application 18177 to P.F. O'Reilly; these data are available through UK Biobank controlled access and cannot be redistributed by the authors. UKB whole genome and exome data used were under the same application and accessed through DNA Nexus. UKB QC resource information was obtained from UKB resource 531. All of Us data are available through application via the All of Us Research Program workbench (controlled access). External summary data used were the UKB exome sequencing results (Backmann et al.2021) and UKB GWAS summary statistics (Neale lab: https://www.nealelab.is/). Population genetic data for investigating theoretical properties of POPout signals under neutrality and stabilising selection were simulated by SLiM-v4.0.

Research involving human participants, their data, or biological material

Policy information about studies with human participants or human data. See also policy information about sex, gender (identity/presentation), and sexual orientation and race, ethnicity and racism.

Reporting on sex and gender	In the UK Biobank, sex is as reported by the NHS at birth unless updated by the participant. In All of Us, sex is reported at birth. For analyses of both data sets, sex was included as a covariate in the trait residualisation step step and the subsequent analyses were performed without regard to sex-specific differences.
Reporting on race, ethnicity, or other socially relevant groupings	UK Biobank population ancestries were defined by 4-means clustering on the first two genotype principal components and then labeled by the majority country of birth (Field 20115) within each cluster. All of Us ancestry categories were as provided by All of Us (gnomAD-aligned labels derived via PCA projection). For the UKB data, the largest ancestry cluster (European) were used for the primary analyses, while one replication analysis was performed in the remaining samples corresponding to the "multi-ancestry" sub-cohort and a further replication was performed in the All of Us data and performed in the European ancestry sub-cohort.
Population characteristics	The UK Biobank (UKB) is a prospective cohort study of approximately 500,000 participants recruited across the United Kingdom from 2006 to 2010, with data available on thousands of biomarkers, anthropometric factors, 'omics measures, imaging variables, questionnaire responses, medications and diseases, both clinically diagnosed and self-reported. The average age of participants at baseline was 55.8 years of age (SD=17.1) and while a large range of ancestries are represented in the cohort, over 80% of the sample are European-ancestry individuals. The All of Us Research Program (All of Us) is a diverse ancestry biobank drawn from across the United States, utilising questionnaire and electronic health record (EHR) data, in a cohort of individuals with a wider age range than the UKB (average age= 55.8, SD=17.1).
Recruitment	Not applicable. Participants were recruited by the UK Biobank and All of Us initiatives; this work is a secondary analysis of those resources under approved applications (UKB app 18177; All of Us workbench access).
Ethics oversight	The analyses performed correspond to secondary analyses of controlled-access human participant data (stored on computer servers)from UK Biobank (approved application 18177) and the All of Us Research Program workbench, for which the authors have approved access. No new recruitment or intervention was performed.

Note that full information on the approval of the study protocol must also be provided in the manuscript.

Field-specific reporting

Please select the one below that is the best fit for your research. If you are not sure, read the appropriate sections before making your selection.

☒ Life sciences ☐ Behavioural & social sciences ☐ Ecological, evolutionary & environmental sciences

For a reference copy of the document with all sections, see nature.com/documents/nr-reporting-summary-flat.pdf

Life sciences study design

All studies must disclose on these points even when the disclosure is negative.

Sample size	Sample sizes were determined by available participants and QC/inclusion criteria in UK Biobank and All of Us datasets; all exact sample sizes are reported in the Methods for each cohort, QC step and analysis. For the traits with least missing data, the total number of samples were approximately 400k in the unrelated UKB European ancestry sub-cohort, approximately 150k in the All of US European ancestry sub-cohort, approximately 50k in the UKB multi-ancestry sub-cohort, and approximately 20k in UKB European ancestry repeated measures sub-cohort, and approximately 20k in the UKB European ancestry sibling pair sub-cohort. Supplementary Table 1 contains exact numbers corresponding to the different traits and tests.
Data exclusions	Standard quality control (QC) procedures were applied independently to each ancestry cluster. SNPs with a minor allele frequency (MAF) <0.01, genotype missingness > 0.02, or Hardy-Weinberg equilibrium test P-value <10−8 were excluded. Samples exhibiting high levels of

	missingness or heterozygosity, inconsistencies in genetic-inferred and self-reported sex, or displaying aneuploidy of the sex chromosomes, were removed in accordance with recommendations from the UKB data processing team (see Methods). For the analyses in the general population, a greedy algorithm was employed to remove related individuals that maximises sample retention while removing all third-degree relatives (kinship coefficient > 0.044). Additional sample exclusions were for diseases and medications with potential effects on the traits, as described in the Methods, as well as trait outliers (6 SD). Traits were selected among three UKB classes (Biomarkers, Physical Measures, Social/Cognitive/Demographic) and were filtered based on sample size (>100k samples post-exclusions), skewness filter, minimum number of distinct values and modal constraints; common-SNP heritability estimate > 5%; oligogenic exclusion (common SNPs that explain > 2% trait variance). Traits with high genetic correlation were removed, retaining the correlated trait with larger sample size. Additionally, traits that failed POPout specific QC (significant POPout in middle 80% of trait distribution) were removed.
Replication	Primary POPout analyses were replicated in three independent cohorts: (1) UKB European-ancestry repeated-measures cohort, (2) UKB multi-ancestry sub-cohort, and (3) All of Us European-ancestry sub-cohort. Replication inclusion requirements included a minimum of 5,000 samples with corresponding genetic and trait data that passed the standard POPout QC, as described in Methods.
Randomization	UK Biobank samples of the primary analyses (Fig.2b) were randomly assigned to the discovery half, in which a GWAS was performed on each trait, and a test half, in which polygenic risk scores (for each trait) were computed and POPout analyses were performed. Rare variant and burden test analyses were performed on the corresponding samples with each of the discovery and test halves. The POPout replication analyses used all available samples from the three replication cohorts (repeated measures and multi-ancestry cohorts of the UKB, and the All of Us cohort)and the already-optimized PRS models to maximize replication sample sizes - these replication cohorts were entirely independent of the samples used in the primary analysis and, thus, did not require randomization. For the sibling analyses, siblings were randomly assigned to index and conditional status (as required by the STANDout test).
Blinding	Not applicable (no intervention and no blinded outcome assessment described).

Reporting for specific materials, systems and methods

We require information from authors about some types of materials, experimental systems and methods used in many studies. Here, indicate whether each material, system or method listed is relevant to your study. If you are not sure if a list item applies to your research, read the appropriate section before selecting a response.

Materials & experimental systems

n/a	Involved in the study
☒	☐ Antibodies
☒	☐ Eukaryotic cell lines
☒	☐ Palaeontology and archaeology
☒	☐ Animals and other organisms
☒	☐ Clinical data
☒	☐ Dual use research of concern
☒	☐ Plants

Methods

n/a	Involved in the study
☒	☐ ChIP-seq
☒	☐ Flow cytometry
☒	☐ MRI-based neuroimaging

Plants

Seed stocks	*Report on the source of all seed stocks or other plant material used. If applicable, state the seed stock centre and catalogue number. If plant specimens were collected from the field, describe the collection location, date and sampling procedures.*
Novel plant genotypes	*Describe the methods by which all novel plant genotypes were produced. This includes those generated by transgenic approaches, gene editing, chemical/radiation-based mutagenesis and hybridization. For transgenic lines, describe the transformation method, the number of independent lines analyzed and the generation upon which experiments were performed. For gene-edited lines, describe the editor used, the endogenous sequence targeted for editing, the targeting guide RNA sequence (if applicable) and how the editor was applied.*
Authentication	*Describe any authentication procedures for each seed stock used or novel genotype generated. Describe any experiments used to assess the effect of a mutation and, where applicable, how potential secondary effects (e.g. second site T-DNA insertions, mosiacism, off-target gene editing) were examined.*
