# Supplementary material for: Distinct genetic architecture in the tails of complex traits
Source: Nature. 2026 May 27;655(8123):676–84. doi: 10.1038/s41586-026-10516-5 (PMC13372694; doi:10.1038/s41586-026-10516-5)
Supplement: Supplementary file 4 — Peer Review File [file 41586_2026_10516_MOESM4_ESM.pdf]

nature portfolio

Peer Review File

Distinct genetic architecture in the tails of complex traits

Corresponding Author: Professor Paul OReilly

This file contains all reviewer reports in order by version, followed by all author rebuttals in order by version.

Version 0:

Reviewer comments:

Referee #1

(Remarks to the Author)
The manuscript by Souaisia et al. presents an analysis of genetic architecture focusing on individuals in the tails of the phenotypic distribution. For 68 out of 74 quantitative traits studied, the authors find that common variants do not accurately predict the phenotypes of individuals in the tails of the distribution. The authors replicate these findings in a separate multi-ancestry cohort of the UK BioBank, the All of Us data, and in a sibling-based analysis, suggesting that these are indeed real biological effects. Instead, the authors hypothesize that individuals in the tails are enriched for rare variants of large effect. These rare variants are not well-captured in the polygenic scores, which is why these individuals' traits were not well-predicted by the polygenic scores. Consistent with their hypothesis, the authors find an enrichment of rare variant associations for the traits with this tail effect. Lastly, the authors show that stabilizing selection can generate this enrichment of large-effect rare variants in individuals at the tails of the phenotypic distribution.

Overall, I found this manuscript to be a highly innovative and creative study of genetic architecture. The systematic focus on the tails of the phenotypic distribution and the development of the POPout test is clever. For the most part, the analyses appear rigorous and the authors did a nice job of replicating the main results in different cohorts and using different approaches. I believe this manuscript will have wide-reaching implications on the study of the architecture of complex traits.

I have a number of comments to improve the manuscript:

1) My biggest overall critique is the initial framing of the present results as not supporting "polygenic architecture" or the infinitesimal model. This can be seen in lines 43-50 of the manuscript. I'm not sure that finding that there are rare variants of large effect on the traits in individuals in the tails of the phenotypic distribution is necessarily inconsistent with a polygenic architecture or the infinitesimal model. Indeed, the authors own simulations show that simulations under the normal quantitative genetics model with stabilizing selection can generate this pattern. The infinitesimal model does not preclude the effects of stabilizing selection, and as such, I don't think is incompatible with the empirical results in this paper. In essence, I think the statement that "our hypothesis diverges from the infinitesimal model to the extent that selection constrains large-effect alleles to remain at low frequencies, effectively concentrating rare alleles in the tails of complex traits." (lines 46-48) is largely based on how one defines the infinitesimal model. Framing the present study around an extreme definition of the infinitesimal model may not be the best approach. Instead, I think it would be stronger if the present study was framed more around the existence "rare variants of large effect" and the presence of stabilizing selection on traits and the consequences of both of these on polygenic scores.

2) Lines 59-61: This part was a little confusing as written.

3) Lines 59-61: Antaki et al. (2022 Nature Genetics) also found that autistic individuals with a higher rare variant load tended to have a lower polygenic score for autism compared to autistic individuals with fewer rare variants. This finding supports the authors' hypothesis of rare variants of large effect leading to departures from phenotypes expected from polygenic scores. Consider citing and discussing this study here.

4) Lines 130-133: This was confusing as phrased. Maybe considered clarifying what "POPout effects in the direction expected…" means.

5) Line 221 (and 620-621: The authors use an evolutionary model of stabilizing selection where selection is applied for 1N generations after a burn-in of 10N generations. Why was stabilizing selection not applied in the burn in? Do the results

change if stabilizing selection is applied for more than 1N generations?

6) Line 614: In the SLIM simulations of stabilizing selection, the authors draw mutation effect sizes from a gamma distribution with shape of 0.186 and a mean of 0.01026. They cite reference 57 for this. While reference 57 does indeed use a gamma distribution for variant effects, the specific parameters used in the authors' simulations are not specified here. So, a better justification for the use of these parameters is needed. Additionally, how sensitive are the results to the distribution of variant effect sizes? Some additional testing of other distributions, potentially with a normal distribution of effect sizes, would strengthen these results.

7) Line 232: The authors write that they "…applied a method for inferring ongoing selection…". While I appreciate that space is limited, a little more description and detail here beyond just "a method" would be good.

8) Line 248: It appears that the authors used the methods described in Sanjak et al. 2018 to detect ongoing selection. Sanjak et al. also did this. How do the authors' present results compare to those found in Sanjak et al. (for the traits that were considered in both studies)?

9) Lines 290-298: I think that the importance of large-effect rare variants for the individuals in the tails of the distribution could be another reason for the poor portability of polygenic scores across genetic ancestry. The rare variants in one ancestry are less likely to be found in individuals that are not closely related. It might be worth mentioning this connection here.

10) Lines 241-242: It would be good to more clearly specify here the intuition behind the expected POPout effects and the selection analysis.

(Remarks on code availability)

Referee #2

(Remarks to the Author)
In this manuscript, Souaiaia et al. use a new approach to demonstrate the enrichment of rare, large-effect genetic variants in the tails of quantitative traits. Their method, POPout, leverages the statistical fact that if two variables X, Y follow a bivariate normal distribution, then E(Y|X) is a linear function of X, and vice versa. Under an interpretation of the polygenic model of a quantitative trait, the value of a trait, Y, exhibits such a relationship with its genetic component, G. POPout tests this model against the alternative that in the extreme tails of Y, the relationship with G (estimated via a PGS) is attenuated; in such cases, a likely interpretation is that extreme values of Y are driven not by any common-variant component but rather by rare variants of large effect. POPout rejects the bivariate normal model for most traits analyzed. The authors frame these results as a major departure from the canonical understanding of polygenic architecture.

Although I have concerns about the framing, POPout itself is a novel and interesting approach. It allows for the detection of rare-variant tail enrichment without even observing those variants, and in principle, it could have been applied to obtain these results before the advent of large-scale sequencing studies. Its results are supported by the group's previously-developed STANDout method, which uses an orthogonal source of data (from sibling pairs) and which also looks for a deviation from gaussianity; in this case, the property being tested is that if X,Y are bivariate normal then Var(Y|X) is constant as a function of X. Using these two approaches, the authors find convincing evidence to support the hypothesis that the tails of complex traits are highly enriched for carriers of rare, large-effect variants.

I have one major comment about framing, one major comment about software, and several minor comments/suggestions.

Major comments
1. I don't agree with the authors that these findings challenge our current understanding of genetic architecture. To some extent 'current understanding' is a moving target, but I'll highlight some previous papers that either anticipate the results of this study, or that demonstrate an understanding of the tail-enrichment phenomenon.

a. As the authors do acknowledge, Fiziev et al. (2023 Science) quantify the enrichment of elevated rare variant PGS in the tails of complex traits. Their approach is perhaps less elegant but more direct, as the enriched variants are actually observed, and the amount of enrichment is quantified in interpretable units (odds ratios). They write:

"Although rare variant PRSs underperformed for average phenotype predictions, we reasoned that they may outperform common variant PRSs for identifying individuals at phenotypic extremes, which is more relevant for clinical screening and risk management. Indeed, individuals with an outlier phenotype (z-score ≥3) were 10-fold more likely than the overall population to have a rare variant PRS score in the 0.1st or 99.9th percentile, compared to 3-fold for common variant PRS (P=0.0026, Fig. 2E,Fig. S6)."

b. Certain studies have sought to identify rare variant associations by specifically detecting extreme phenotype values, because they expected to see a strong tail enrichment. From Xin Li et al. 2017 Nature,

"Here, … we identify gene expression outliers, or individuals showing extreme expression levels for a particular gene, across 44 human tissues. We find that 58% of underexpression and 28% of overexpression outliers have nearby conserved

rare variants compared with 8% of non-outliers."

c. This phenomenon is also known – probably, best known – in the context of common disease, where severity varies significantly. From Zoghbi et al. 2021 PNAS:

"We compared the burden of rare, damaging missense and loss-of-function variants between severe, extremely treatment-resistant schizophrenia, typical schizophrenia, and controls... [We observed] 8.9% of individuals with severe, extremely treatment-resistant schizophrenia carrying a damaging missense or loss-of-function variant compared to 2.3% of typical schizophrenia (odds ratio, 5.48; 95% CI, 1.52 to 19.74; P = 0.02) and 1.6% of controls (odds ratio, 5.82; 95% CI, 3.00 to 11.28; P = 2.6 × 10-8). "

d. It is also known for non-gene expression quantitative traits; from Peloso et al. 2016 EJHG:

"We demonstrate that the power gain for an extreme phenotypic selection study design is much greater in rare variant studies than for studies of common variants. "

In sum, it's already clear that rare variants can have large effects on QTs, that as a result they are highly enriched in the tails, and moreover that they are more tail-enriched as compared with the polygenic common variant component. I think the manuscript should be re-framed as a novel approach to investigate a known phenomenon.

2. The published software needs a lot of work. Currently all the code seems to be in a few giant scripts with no documentation, not even a readme, and I have no idea where I'd start if I wanted to run POPout myself. The Code Availability section promises that there is an R package, which doesn't seem to exist.

Minor comments
3. The section on selection was interesting, but an important missing variable is the mutational target size for large-effect alleles. A trait can be under strong stabilizing selection yet lack rare variants of large effect simply because such alleles never arise. So as far as the implications of POPout for evolutionary parameters, I'd say it tells us more about mutational targets – which traits are able to have mutations of large effect, in which directions? – than it does about selection per se, as we already know that large-effect alleles are generally subject to selection.

4. The comparison between POPout effects and contemporary selection inferred from fecundity seems particularly interesting, but I was confused about the "linear", "disruptive", and "stabilizing" labels in Figure 4g; what do these refer to?

5. I recommend some kind of comparison between the magnitude of POPout effects with what is expected given known rare-variant associations. One way to do this would be to add to the common-variant PGS a rare-variant PGS comprising known associations – does the POPout effect persist? Does the remaining effect seem to be consistent with estimates of the burden heritability (Weiner & Nadig et al. 2023 Nature; I am senior author)? This analysis would be interesting because if there are a lot of rare variant effects that we don't currently observe (e.g., hard-to-type SVs), then POPout effects should be larger than predicted from exome data.

6. Because both POPout and STANDout test for deviations from bivariate normality, it is an important step of the analysis that the phenotype values are quantile normalized. A potential concern is that this normalization would cause conservative bias in POPout effects, because it might bring extreme phenotype values closer to the mean. I recommend mentioning this normalization step in the main text and either providing some discussion of how it may or may not cause bias, or performing a simulation study.

7. The POPout statistic is defined on line 497 as the mean difference between the absolute value of two quantities. This is somewhat confusing because it is informative when the sign of the PRS is different from the sign of the phenotype. What if you instead compute the mean value of:
sgn(y_i) (f(y_i) - PRS_i)
which would still have the desired effect that a positive statistic means regression to the mean. I think this statistic would have the same exact interpretation but have greater statistical power.

8. I was confused by the description of the PRS analysis (l. 426-436). Is POPout applied to the same individuals as were used to construct the PRS, or does "overfit" just refer to the p-value selection procedure? If the former, I recommend mentioning this in the main text and justifying why it isn't an issue. If the latter, I recommend clarifying this text.

9. l. 237: you mean "limited snapshot"?

Signed,
Luke O'Connor

(Remarks on code availability)
The published code is completely inadequate, with no documentation whatsoever, and I cannot even find where POPout is implemented.

Referee #3

(Remarks to the Author)

This manuscript is interesting in that it focuses on the behaviors at the interesting tails of trait distribution, which is arguably most relevant for diseases in contrast to the normal healthy range of traits. The authors point out the interesting distribution range of traits that is closer to Mendelian diseases rather than complex diseases. Although interesting, the concepts are not brand new and the importance of identifying and studying rarer alleles with larger effects has already been appreciated since about two decades ago. The progress has been largely limited by sample ascertainment and sequencing resources for certain population groups. The authors have carried out careful analyses but some of the analyses are simplistic and somewhat repetitive.
I also have quite a few specific questions and comments.
Page 4, lines 111-112, QC step for “the middle 80 trait centiles”: how many traits were removed based on this criterion?
Page 4, line 114: I am also curious to see how results would vary with different definitions/thresholds of extreme values, especially given some complex diseases have much higher prevalence than 1%.
Page 6, line 134: The “large, non-Gaussian, unadjusted environmental risk factors” could also offer a potential explanation of why most trait tails demonstrate departure from polygenicity beyond the offered hypothesis of rare allele enrichment. It's possible that large unadjusted environmental factors drive the trait values toward the tails and they cannot be captured by PRS, resulting in the observations that tail trait values may show PRS towards the mean. This potential explanation undermines the value of the central hypothesis proposed here: primarily due to rarer variants with larger effect sizes.
Page 6, line 139: “disease prevalence of 1%” It's by nature more difficult for PRS to predict rarer yet polygenic diseases. Is this pattern also observed for traits with tails not demonstrating departure from polygenicity, e.g., phosphate, as a negative control mentioned by the authors? I am also wondering if under-powered PRS performance for some complex diseases could be explained by this tail departure. As the authors showed in the dichotomized tail behaviors, top 1% showed poor performance while top 25% showed expected performance. Along with this, it would be interesting to more systematically evaluate the relationship between PRS performance for complex diseases and their estimated prevalence to see if there is any relationship.
Page 6, line 156: It seems the authors performed UKB Multi-Ancestry analysis combing all the ancestries together. Do the authors consider ancestry as a potential confounder of tail behaviors, especially given some traits have different mean, variance and extreme values across ancestries, and rich literatures have shown PRS had varied performance across ancestries? I would suggest performing replications separately for each population, perhaps in AoU, which has larger non-EUR sample sizes than UKB.
Line 199: The authors hypothesized that one explanation of trait tails demonstrating departure from polygenicity is enrichment of rare alleles in the tails. They further used WES associations from a previous study to validate their hypothesis, where the results show significant but modest correlation between the amount of tail departure and the number of WES signals. Given that UKB has WGS data available now, it would be much more compelling to validate with WGS associations. In addition, the authors only looked at associations between number of WES signals and amounts of tail departure. It would be more interesting to see if such departure could be mitigated after accounting for these rare alleles to further confirm.
Line 245: “proxy measures of fitness”: It would be helpful to provide more intuitions of why such associations are expected and why these outcomes are selected as proxy measures of fitness.
Line 354: It seems the authors only used ~800K genotyped markers without considering imputed data. I am wondering if the tail departure could be explained or mitigated by untyped common variants.
Line 361: The authors applied MAF 1% to calculate PRS for common variants. While 1% is a commonly used cutoff for common variants, given the large sample size of UKB, many PRS analyses in literature went beyond the thresholds to capture more signals. I am wondering how the results would change if relaxing the threshold, e.g., to 0.1% to include some low frequency variants in PRS. It may strengthen the claim that rare allele enrichment leads to tail departure from polygenicity if such trend was mitigated.
Line 379: a distribution would be helpful to make sure there are not a lot of pairs at IBS0 above but close to the threshold.
Line 579: Only using BIC to select models may correspond to two scenarios: (1) the selected model is much more favored over others; (2) the selected model performs very similar to another model but is randomly selected. Thus, I would suggest some sensitivity analyses: for each trait, recording the two models with highest BICs, perform a likelihood ratio test to see if the final model (higher BIC between the two) is significantly better than the other one.

(Remarks on code availability)

Version 1:

Reviewer comments:

Referee #1

(Remarks to the Author)
The authors did an excellent job with revising their manuscript. In particular, they have added a number of new and substantial analyses which buttress their original claims. They have also performed some robustness checks on a number of key analyses. Lastly, the framing of the paper is much improved to be better in line with the existing literature.

I believe this is an important study, worthy of publication.

That said, I have a number of suggestions on things to improve. Many of these comments stem from the new analyses that the authors included in the revised manuscript.

1. Title: Overall, the authors did a nice job with framing the manuscript better in terms of the existing literature. The current title now reads, “Striking departures from common polygenicity in the tails of complex traits”. I’m afraid that this phrasing seems awkward and ambiguous to me. My particular unease stems from the “Common polygenicity” phrase. I would suggest the authors try to find different words to say what they really mean, rather than leaning on the jargony “polygenicity”. Some alterative titles could be, “Striking departures from common variant architectures in the tails of complex traits” or “Large-effect rare alleles drive the tails of complex traits”.

2. Line 8 (and elsewhere): Similar to my comment above, I suggest using something other than “common polygenicity” to describe this phenomenon of rare alleles of large effect influencing phenotypes in the tails.

3. Line 41: “…contrasting expectations under neutrality…”. “Expectations” by itself is a little awkward. Expectations of what? Consider rephrasing.

4. Lines 246-249 and Figure 4d: I had a really difficult time following most aspects of the rare variant heritability analysis. Lines 246-248 outlines the results for the heritability calculated using only common variants and for the heritability accounting for non-genetic factors. What about when including rare variants? That result isn’t mentioned din the text here. I thought this would be the main result, so its absence in the Results is a little odd.

5. Figure 4d: Related to my comment above, if I understand things correctly, it appears that the heritability estimates are slightly lower when including the rare variants compared to when only using common variants. This would seem to go opposite what I would expect to be happening under the authors’ hypothesis. Thus, I am concerned that this heritability analysis includes some unrealistic assumptions or is biased in some other way.

6. Figure 5e and associated discussion: Testing for an association of the POPout scores and particular traits related for fitness is clever. However, the description of this analysis provided in the legend for Figure 5e, the main results, and even the Methods section is lacking in clarity and details. Some of the confusion, I think, stems from stating (e.g. lines 808-809) that the “mean value was calculated separately in the upper and lower tail for every trait.” It’s not clear what “trait” refers to here. If I’m understanding this, the authors took the upper tail of one of the 5 fitness traits, and then computed the mean value for each of the 74 other UKBB traits that they computed POPout on. They then tested whether the POPout static for the 74 UKBB traits was correlated with differences in that trait co-varying with differences in fitness. Another question is how the “lower and upper tail means” were tested for the correlation with POPout. How was the difference in means between the tails tested? Or the lower and upper tails separately? Why? More description is needed. Maybe a diagram could help as well.

7. There are a number of related references that would be good to include regarding selection acting on complex traits decreasing the portability of PRS. These include: PMID: 40499537, PMID: 33691092, PMID: 35430887, PMID: 35522704.

(Remarks on code availability)

Referee #2

(Remarks to the Author)
In their revised manuscript, Souaiaia et al. reframe their findings around the following hypothesis:

>Based on the existence of rare alleles that can alone generate extreme trait values, and growing evidence that stabilising selection is a pervasive driver of complex trait genetic architecture, we hypothesise that stabilising selection on some traits may be strong enough to have produced marked enrichments of high-effect, rare alleles in the tails of continuous, complex traits.

This addresses my previous concern that tail enrichment is a known phenomenon which does not contradict existing models. Also, the authors have thoroughly addressed my comment about code availability.

I still have major comments about the revised manuscript. Comments 1-2 are related to the new framing, and comment 3 is related to the new heritability analysis.

Major comments
1. A subtle point bothered me when reading the revised manuscript. We know that trait-associated variants are under negative selection, and we know this because large effect alleles are rare. The rarity of large effect alleles is what causes tail enrichments. When the authors state that tail enrichments are caused by negative selection, this is true, but there is no causal arrow from negative selection to tail enrichment that does not go through allele frequencies – and allele frequencies are themselves the evidence we have for selection. All believable models of selection on traits already predict that large-effect alleles are rare and would therefore also predict tail enrichment. On the other hand, it is still of interest to identify novel consequences of selection even if they do not help us to distinguish between competing models. I suggest that when

introducing the hypothesis that selection explains tail enrichments, the authors explicitly lay out that selection directly affects allele frequencies, and it is the rarity of large-effect alleles that causes tail enrichments.

2. Relatedly, now that selection is a central theme of the paper, the authors should be careful about different models of selection. In the Introduction, the authors frame their hypothesis around a particular model, direct stabilizing selection, and state that this model fits their data better than a neutral model (this is convincing). Later on, they show that alternative models involving directional selection fit their data better for particular traits. I do not really recommend repeating the simulations under these various models. Instead, I recommend editing the text in two ways: (1) to clarify that there exist various modes of selection that could produce similar results; and (2) to distinguish between selection on trait-associated alleles vs. selection on traits (i.e., pleiotropic vs direct selection). I don't think the current handling of pleiotropy in the Discussion really helps with this point.

3. The new calculation of h2_her is interesting, but the estimates are implausible.
They are definitely too large – I think if you compared them with common variant estimates, they would imply rare h2 >> common h2 for many traits, and probably they would imply common h2 + rare h2 >> twin h2. Worse, it looks to me like the largest estimates correspond to the least heritable/least powered traits, such as glucose levels (non-fasting glucose is famously noisy) and age at menopause (which has 50% smaller sample size). Height, definitely the best-powered and most-heritable, has a much smaller estimate; but for some reason, sitting height has a much larger estimate.

The procedure that estimates h2_her is intricate, and I can see any number of reasons that it could fail. My guess would be that statistical noise in the POPout statistics causes bias in the heritability estimate. I recommend either dropping this estimator from the manuscript or performing extensive validation + troubleshooting analyses.

This analysis was intended to address my previous question about whether POPout effects are explained by known rare variant effects, in particular the burden heritability. I liked the analysis where the authors conditioned on a rare-variant PRS. I recommend expanding this analysis to include exome-wide significant burden statistics; I note that for many traits, such as height and LDL, roughly half of burden heritability is explained by exome-wide significant genes that can be included in a PGS.

Minor comments
4. In derivation of the h2^her estimator, the authors skip a key step in the derivation between equation (2) and the following equation – it looks like there are two terms, one involving y and the other involving y', which are assumed to cancel out. I believe that this is actually correct but non-obvious, and it should be spelled out and justified if this estimator is retained in the text.

5. I should have flagged this before; "positive selection" and "negative selection" on traits should be called "positive directional selection" and "negative directional selection" ("positive selection" being a type of selection that acts on an allele).

6. The new Discussion sentences about the mutational target size somewhat miss the point of my previous comment. Instead of being a limitation that target size is not considered, I'd call it a missed opportunity: suppose we already knew that large-effect alleles can only be rare, but we didn't know if they can exist at all. Maybe there is no mutation in the genome that causes a 10cm effect on height. POPout would tell us that such mutations can indeed occur; it tells us something potentially new about the mutational target.

7. Regarding training the PGS and running POPout in the same individuals, is it true that this is done in UK Biobank but not in 'UKB multi-ancestry' and AoU?

Signed,
Luke O'Connor

(Remarks on code availability)

Referee #3

(Remarks to the Author)
The authors have made pains-taking efforts to address all my previous comments.

(Remarks on code availability)
The codes are reasonably easy to follow but I have not tested the codes myself.

Version 2:

Reviewer comments:

Referee #1

(Remarks to the Author)
The authors have addressed all of my concerns on the previous versions of the manuscript. I think the manuscript is much-

improved and I believe it represents and important and significant contribution.

(Remarks on code availability)

Referee #2

(Remarks to the Author)
The manuscript is significantly improved, and I have no remaining comments.

(Remarks on code availability)

Open Access This Peer Review File is licensed under a Creative Commons Attribution 4.0 International License, which permits use, sharing, adaptation, distribution and reproduction in any medium or format, as long as you give appropriate credit to the original author(s) and the source, provide a link to the Creative Commons license, and indicate if changes were made.
In cases where reviewers are anonymous, credit should be given to 'Anonymous Referee' and the source.
The images or other third party material in this Peer Review File are included in the article’s Creative Commons license, unless indicated otherwise in a credit line to the material. If material is not included in the article’s Creative Commons license and your intended use is not permitted by statutory regulation or exceeds the permitted use, you will need to obtain permission directly from the copyright holder.
To view a copy of this license, visit https://creativecommons.org/licenses/by/4.0/

Referees' comments:

Referee #1 (Remarks to the Author):

The manuscript by Souaiaia et al. presents an analysis of genetic architecture focusing on individuals in the tails of the phenotypic distribution. For 68 out of 74 quantitative traits studied, the authors find that common variants do not accurately predict the phenotypes of individuals in the tails of the distribution. The authors replicate these findings in a separate multi-ancestry cohort of the UK BioBank, the All of Us data, and in a sibling-based analysis, suggesting that these are indeed real biological effects. Instead, the authors hypothesize that individuals in the tails are enriched for rare variants of large effect. These rare variants are not well-captured in the polygenic scores, which is why these individuals' traits were not well-predicted by the polygenic scores. Consistent with their hypothesis, the authors find an enrichment of rare variant associations for the traits with this tail effect. Lastly, the authors show that stabilizing selection can generate this enrichment of large-effect rare variants in individuals at the tails of the phenotypic distribution.

Overall, I found this manuscript to be a highly innovative and creative study of genetic architecture. The systematic focus on the tails of the phenotypic distribution and the development of the POPout test is clever. For the most part, the analyses appear rigorous and the authors did a nice job of replicating the main results in different cohorts and using different approaches. I believe this manuscript will have wide-reaching implications on the study of the architecture of complex traits.

I have a number of comments to improve the manuscript:

1) My biggest overall critique is the initial framing of the present results as not supporting "polygenic architecture" or the infinitesimal model. This can be seen in lines 43-50 of the manuscript. I'm not sure that finding that there are rare variants of large effect on the traits in individuals in the tails of the phenotypic distribution is necessarily inconsistent with a polygenic architecture or the infinitesimal model. Indeed, the authors own simulations show that simulations under the normal quantitative genetics model with stabilizing selection can generate this pattern. The infinitesimal model does not preclude the effects of stabilizing selection, and as such, I don't think is incompatible with the empirical results in this paper. In essence, I think the statement that "our hypothesis diverges from the infinitesimal model to the extent that selection constrains large-effect alleles to remain at low frequencies, effectively concentrating rare alleles in the tails of complex traits." (lines 46-48) is largely based on how one defines the infinitesimal model. Framing the present study around an extreme definition of the infinitesimal model may not be the best approach. Instead, I think it would be stronger if the present study was framed more around the existence "rare variants of large effect" and the presence of stabilizing selection on traits and the consequences of both of these on polygenic scores.

>> We agree with the reviewer that it was not best to frame this work as inconsistent with the infinitesimal model, since modern interpretation of the model (often now described as the "polygenic model") does allow for the effects of stabilizing selection and large-effect, rare alleles. We have now removed the section relating to the infinitesimal model and changed the framing of the paper entirely (title, abstract and intro). The new framing is built around our Figure 1, which – reflecting the reviewer's proposal – assumes the existence of rare variants of large effect when traits are under stabilising selection, and sets up the study to focus on investigating *the extent to which* selection may have produced enrichments of rare variants in the tails of different traits (depending on selection strength/type), as well as the multiple consequences of this (e.g. on observed genetic architecture, the

accuracy of polygenic scores and their prediction of disease, the distribution of trait values within families, and consequences for rare variant mapping studies). We thank the reviewer for making this point and we believe that the framing of the paper is now far clearer as a result.

2) Lines 59-61: This part was a little confusing as written.

>> This has now been reworded.

3) Lines 59-61: Antaki et al. (2022 Nature Genetics) also found that autistic individuals with a higher rare variant load tended to have a lower polygenic score for autism compared to autistic individuals with fewer rare variants. This finding supports the authors' hypothesis of rare variants of large effect leading to departures from phenotypes expected from polygenic scores. Consider citing and discussing this study here.

>> We thank the reviewer for pointing out this study, which we have now highlighted as supporting our observations in the Discussion.

4) Lines 130-133: This was confusing as phrased. Maybe considered clarifying what "POPout effects in the direction expected…" means.

>> We agree that this section was not clearly phrased and so we have now reworded that paragraph to improve clarity.

5) Line 221 (and 620-621: The authors use an evolutionary model of stabilizing selection where selection is applied for 1N generations after a burn-in of 10N generations. Why was stabilizing selection not applied in the burn in? Do the results change if stabilizing selection is applied for more than 1N generations?

>> We implemented a classical forward-in-time simulation model with a standard neutral burn-in phase, followed by a separate phase under stabilizing selection. Stabilizing selection was not applied during the burn-in to ensure that expected levels of genetic diversity, linkage disequilibrium etc under neutrality are generated from the homogenous population of sequences at initiation that had zero diversity, to allow testing of hypothetical traits under neutrality. We ran 10N generations under neutrality, which should be sufficient to reach an equilibrium (Chadeau-Hyman et al 2008) and generate sequences with population genetic metrics expected under neutrality and so that initial conditions are 'forgotten'. We have now clarified these points in the Methods.

Moreover, following the reviewer's suggestion, we repeated our simulations applying stabilizing selection for more than 1N generations – for both 5N and 10N generations – and observed similar results in relation to tail architecture stabilizing. New results on these extended simulations of stabilizing selection are presented in Supplementary Figures 5 – 7 – summarized and referred to in the main text.

6) Line 614: In the SLIM simulations of stabilizing selection, the authors draw mutation effect sizes from a gamma distribution with shape of 0.186 and a mean of 0.01026. They cite reference 57 for this. While reference 57 does indeed use a gamma distribution for variant effects, the specific parameters used in the authors' simulations are not specified here. So, a better justification for the use of these parameters is needed. Additionally, how sensitive are the results to the distribution of variant effect sizes? Some additional testing of other distributions, potentially with a normal distribution of effect sizes, would strengthen these results.

>> We agree with the reviewer that additional testing to investigate the sensitivity of results to the distribution of effect sizes would strengthen our simulation results substantially. We have now performed extensive additional simulations to test the impact of effect sizes drawn from three different gamma distributions, with low (0.005), medium (0.01, as previously) and high mean (0.02) values, and correspondingly low, medium and high variances (with a 16-fold difference in variance between low and high), as well as a standard Gaussian distribution. The results based on these new simulations of different effect-size distributions also show enrichments of high-effect, rare alleles in the tails – shown in Supplementary Figures 5 and 6 – and corresponding POPout effects as in our original simulations – shown in Supplementary Figure 7. The rare variant tail enrichments and POPout effects are markedly less pronounced for Gaussian effects as expected, since in this case few alleles have distinctly larger effects than most – but, for this reason, a Gamma distribution of effect sizes is considered more realistic for genetic effects (with existence of many known e.g. Mendelian effects). These new simulation sensitivity results are summarized and referred to in the main text.

7) Line 232: The authors write that they "…applied a method for inferring ongoing selection…". While I appreciate that space is limited, a little more description and detail here beyond just "a method" would be good.

>> We have now added a description of the method within the main text to provide greater context to the reader regarding the method used here.

8) Line 248: It appears that the authors used the methods described in Sanjak et al. 2018 to detect ongoing selection. Sanjak et al. also did this. How do the authors' present results compare to those found in Sanjak et al. (for the traits that were considered in both studies)?

>> We have now performed a comparison with the Sanjak et al. analysis for those traits that are overlapping between the studies. Despite differences in sample QC, the different approaches taken to determining the type of inferred selection (e.g. we applied the BIC to select the best-fitting model), and the fact that Sanjak et al performed sex-stratified analyses versus our sex-pooled analyses, the two sets of results are extremely similar, as shown in Supplementary Table 5.

9) Lines 290-298: I think that the importance of large-effect rare variants for the individuals in the tails of the distribution could be another reason for the poor portability of polygenic scores across genetic ancestry. The rare variants in one ancestry are less likely to be found in individuals that are not closely related. It might be worth mentioning this connection here.

>> This is an excellent point and we have now added a section relating to the impact of our findings on portability problems in the Discussion. We highlight both the point that the reviewer makes about the localisation of rare alleles, as well as an additional point about the potential impact of our observations alongside differing diagnostic thresholds of disease across populations. Even with the degree of rare variant tail enrichment fixed across populations, different diagnostic thresholds of disease could lead to different predictive accuracy of common-variant PRS derived in the different ancestral populations given the different mix of common and rare alleles among cases. Portability is an important focus of our own work (PMID: 38123642 and R01HG012773) and we have discussed these issues at length and so it was rather an omission that we did not include discussion of this previously – we are extremely grateful to the reviewer for highlighting this as our Discussion is now significantly improved with this addition.

10) Lines 241-242: It would be good to more clearly specify here the intuition behind the expected POPout effects and the selection analysis.

>> We have now provided the intuition for comparing the POPout and selection analysis results.

Referee #2 (Remarks to the Author):

In this manuscript, Souaiaia et al. use a new approach to demonstrate the enrichment of rare, large-effect genetic variants in the tails of quantitative traits. Their method, POPout, leverages the statistical fact that if two variables X, Y follow a bivariate normal distribution, then E(Y|X) is a linear function of X, and vice versa. Under an interpretation of the polygenic model of a quantitative trait, the value of a trait, Y, exhibits such a relationship with its genetic component, G. POPout tests this model against the alternative that in the extreme tails of Y, the relationship with G (estimated via a PGS) is attenuated; in such cases, a likely interpretation is that extreme values of Y are driven not by any common-variant component but rather by rare variants of large effect. POPout rejects the bivariate normal model for most traits analyzed. The authors frame these results as a major departure from the canonical understanding of polygenic architecture.

Although I have concerns about the framing, POPout itself is a novel and interesting approach. It allows for the detection of rare-variant tail enrichment without even observing those variants, and in principle, it could have been applied to obtain these results before the advent of large-scale sequencing studies. Its results are supported by the group’s previously-developed STANDout method, which uses an orthogonal source of data (from sibling pairs) and which also looks for a deviation from gaussianity; in this case, the property being tested is that if X,Y are bivariate normal then Var(Y|X) is constant as a function of X. Using these two approaches, the authors find convincing evidence to support the hypothesis that the tails of complex traits are highly enriched for carriers of rare, large-effect variants.

I have one major comment about framing, one major comment about software, and several minor comments/suggestions.

Major comments
1. I don’t agree with the authors that these findings challenge our current understanding of genetic architecture. To some extent ‘current understanding’ is a moving target, but I’ll highlight some previous papers that either anticipate the results of this study, or that demonstrate an understanding of the tail-

enrichment phenomenon.

a. As the authors do acknowledge, Fiziev et al. (2023 Science) quantify the enrichment of elevated rare variant PGS in the tails of complex traits. Their approach is perhaps less elegant but more direct, as the enriched variants are actually observed, and the amount of enrichment is quantified in interpretable units (odds ratios). They write:
"Although rare variant PRSs underperformed for average phenotype predictions, we reasoned that they may outperform common variant PRSs for identifying individuals at phenotypic extremes, which is more relevant for clinical screening and risk management. Indeed, individuals with an outlier phenotype (z-score ≥3) were 10-fold more likely than the overall population to have a rare variant PRS score in the 0.1st or 99.9th percentile, compared to 3-fold for common variant PRS (P=0.0026, Fig. 2E,Fig. S6)."

b. Certain studies have sought to identify rare variant associations by specifically detecting extreme phenotype values, because they expected to see a strong tail enrichment. From Xin Li et al. 2017 Nature, "Here, … we identify gene expression outliers, or individuals showing extreme expression levels for a particular gene, across 44 human tissues. We find that 58% of underexpression and 28% of overexpression outliers have nearby conserved rare variants compared with 8% of non-outliers."

c. This phenomenon is also known – probably, best known – in the context of common disease, where severity varies significantly. From Zoghbi et al. 2021 PNAS:
"We compared the burden of rare, damaging missense and loss-of-function variants between severe, extremely treatment-resistant schizophrenia, typical schizophrenia, and controls... [We observed] 8.9% of individuals with severe, extremely treatment-resistant schizophrenia carrying a damaging missense or loss-of-function variant compared to 2.3% of typical schizophrenia (odds ratio, 5.48; 95% CI, 1.52 to 19.74; P = 0.02) and 1.6% of controls (odds ratio, 5.82; 95% CI, 3.00 to 11.28; P = 2.6 × 10-8). "

d. It is also known for non-gene expression quantitative traits; from Peloso et al. 2016 EJHG:
"We demonstrate that the power gain for an extreme phenotypic selection study design is much greater in rare variant studies than for studies of common variants. "

In sum, it's already clear that rare variants can have large effects on QTs, that as a result they are highly enriched in the tails, and moreover that they are more tail-enriched as compared with the polygenic common variant component. I think the manuscript should be re-framed as a novel approach to investigate a known phenomenon.

>> We agree with the reviewer that it wasn't the right framing to suggest that our results "challenge the current understanding" of genetic architecture – in particular, we should not have suggested that our results conflict with the infinitesimal model, since most contemporary interpretation of the model (or related 'polygenic model') allows for large-effect variants and selection. We also agree that our results may have been anticipated by the results of previous studies, but we do think that those studies did not elaborate on, or specifically investigate, the *degree* of rare variant tail enrichment across a broad range of quantitative traits, nor investigate or test *how selection* contributes to this. We note that multiple traits showed no such enrichment, while others showed only strong enrichment in one and not the other tail, which we believe has not been highlighted, described or investigated before.

Accounting for these points, we have now changed the framing of the paper entirely (title, abstract, intro etc). The new framing excludes the section relating to the infinitesimal model and makes no allusion to challenging the current understanding of genetic architecture. Our new framing is instead

built around Figure 1, which assumes the existence of rare variants of large effect when traits are under stabilising selection, and sets up the study to focus on investigating *the extent to which* selection may have contributed to enrichments of rare variants in the tails of different traits (depending on selection strength/type), as well as the multiple consequences of this (e.g. on observed genetic architecture, the accuracy of polygenic scores and their prediction of disease, the distribution of trait values within families, and consequences for rare variant mapping studies).

We thank the reviewer for these points and references and believe that the framing of the manuscript is now far more accurate as a result.

2. The published software needs a lot of work. Currently all the code seems to be in a few giant scripts with no documentation, not even a readme, and I have no idea where I'd start if I wanted to run POPout myself. The Code Availability section promises that there is an R package, which doesn't seem to exist.

>> We apologise for the state of our code on initial submission. We have now produced a user-friendly software written in Python and R to allow users to run our POPout test on their own data. We have built a new dedicated website (www.tailstests.org) to host our software, which provides download links and clearly describes required packages, user options, input file formatting and output files. The website includes a quickstart guide and simulated toy data from five different traits so that users can begin running POPout immediately.

Minor comments

3. The section on selection was interesting, but an important missing variable is the mutational target size for large-effect alleles. A trait can be under strong stabilizing selection yet lack rare variants of large effect simply because such alleles never arise. So as far as the implications of POPout for evolutionary parameters, I'd say it tells us more about mutational targets – which traits are able to have mutations of large effect, in which directions? – than it does about selection per se, as we already know that large-effect alleles are generally subject to selection.

>> We thank the reviewer for this point about what our results might tell us about mutational targets. We have now described this as a limitation in the Discussion. We note that the mutational target size of high-effect rare alleles is likely a (positively correlated) function of strength of selection, since selective constraint shifts high-effect alleles to low frequency, and what a 'high-effect' allele is is dependent on trait variance, which is also dependent on selection. Thus, we would still expect selection to be the key driver of our observations, but agree that mutational target size is an important missing variable from our study, which should have been previously included in our limitations.

4. The comparison between POPout effects and contemporary selection inferred from fecundity seems particularly interesting, but I was confused about the "linear", "disruptive", and "stabilizing" labels in Figure 4g; what do these refer to?

>> These refer to the different types of selection inferred by the final model – specifically the quadratic term (gamma) – of the Sanjak method. We have now changed the "linear" label, which was insufficiently informative about selection, to "Directional Only" (meaning no quadratic term in the final model,

gamma=0). This should help to make the other terms clearer, the meaning of which we have now described in greater detail in the caption of the figure (now Fig.5).

5. I recommend some kind of comparison between the magnitude of POPout effects with what is expected given known rare-variant associations. One way to do this would be to add to the common-variant PGS a rare-variant PGS comprising known associations – does the POPout effect persist? Does the remaining effect seem to be consistent with estimates of the burden heritability (Weiner & Nadig et al. 2023 Nature; I am senior author)? This analysis would be interesting because if there are a lot of rare variant effects that we don't currently observe (e.g., hard-to-type SVs), then POPout effects should be larger than predicted from exome data.

>> To address this point and some of those from reviewer 3, we undertook novel rare variant analyses using the recently available WGS data in the UK Biobank (UKB). We first performed variant-by-variant association testing among rare variants down to a MAF of $1x10^{-4}$ across all 74 QC'ed traits and in the same half of the UKB sample as previously used for common-variant GWAS. Next, we aggregated the significant variants from these analyses to compute a rare-variant PRS for each trait in the other (test) half of the UKB and added this to our existing common-variant PRS. We then evaluated any reduction in the POPout signal from inclusion of these 'intermediate' rare variants. This approach constitutes a highly tractable, replicable and robust way to incorporate rare variants into our PRS and test for any reduction in POPout effects. Our results show that among 68 trait tails with significant POPout and a corresponding rare PRS for testing (i.e. with at least one significant rare variant), 49 had reduced POPout ($P = 6.5x10^{-5}$), 7 had significantly reduced POPout with a mean reduction in POPout effect of 27% and 0 had significantly increased POPout (Fig.4d). While the inclusion of these rare variants in the PRS increased trait variance explained by the PRS only modestly (5.4%), the increase in odds ratio relating to extreme trait status is substantial (16.5%; Supplementary Table 2), confirming the importance of rare variants in prediction of extreme trait values. Moreover, a large fraction of the rare variants that we identified and included in the rare PRSs were present in the ClinVar database (67 of 609 unique variants; see Supplementary Table 4), providing reassurance that our WGS analyses were robust and identified variants with established effects on the relevant traits.

In relation to the remaining effect, we derived an analytical approach (see Methods) for estimating the heritability explained by high-effect rare alleles (which we denote h^2_{HER}) that we report before and after incorporating rare variants and additionally after assuming that the contribution of rare variants is proportional to the trait heritability (with non-genetic factors explaining the rest of the signal). Across all 68 trait tails before incorporating rare variants, mean h^2_{HER} = 0.012 (range 0.031 – 0.39; minus early age-at-menopause), and after assuming non-genetic contribution to POPout effects, mean h^2_{HER} = 0.069 (range 0.0056 – 0.21; minus early age-at-menopause). These estimates are somewhat higher than the "burden heritability" estimates of Weiner & Nadig et al, although this may be explained by our estimates incorporating every class (including hard-to-type) of variant genome-wide, rather than only gene-wise coding variants, as the reviewer suggests.

The new WGS rare variant and POPout recovery analyses are described in the main text and presented in a new Figure 4 that focuses on the WES, WGS, POPout recovery and h^2_{HER} estimates.

6. Because both POPout and STANDout test for deviations from bivariate normality, it is an important step of the analysis that the phenotype values are quantile normalized. A potential concern is that this normalization would cause conservative bias in POPout effects, because it might bring extreme phenotype values closer to the mean. I recommend mentioning this normalization step in the main text and either providing some discussion of how it may or may not cause bias, or performing a simulation study.

>> The inverse normal transformation step was performed to increase robustness of the subsequent linear regression that the POPout test is based on. As the reviewer suggests, this does result in conservative bias in the POPout test, although we think that this is necessary to guard against potential inflation of the statistic caused by non-normality of residuals in the test regression. We have now highlighted and justified this normalisation step in the main text.

7. The POPout statistic is defined on line 497 as the mean difference between the absolute value of two quantities. This is somewhat confusing because it is informative when the sign of the PRS is different from the sign of the phenotype. What if you instead compute the mean value of:
sgn(y_i) (f(y_i) - PRS_i)
which would still have the desired effect that a positive statistic means regression to the mean. I think this statistic would have the same exact interpretation but have greater statistical power.

>> This was in fact the statistic that we used in our original code and, thus, testing, but our statistic was not written correctly in the text. So results remain the same but we have corrected the equation in the text.

8. I was confused by the description of the PRS analysis (l. 426-436). Is POPout applied to the same individuals as were used to construct the PRS, or does “overfit” just refer to the p-value selection procedure? If the former, I recommend mentioning this in the main text and justifying why it isn’t an issue. If the latter, I recommend clarifying this text.

>> POPout is applied to the same individuals as were used to construct the PRS. This was done to optimise the sample size for POPout testing, since the POPout test relies on the observed PRS in only 1% of the sample. We consider this justified because (1) the degree of overfitting should be limited as we only optimise the P-value threshold among 10 thresholds of typically highly correlated PRSs, and (2) because this approach is conservative, in that optimising the PRS would tend towards reduced POPout, thus producing a slightly deflated, rather than inflated, statistic. We previously explained this in the Methods section (now clarified further), but we agree with the reviewer that this should be highlighted and justified in the main text, which we have now done.

9. l. 237: you mean “limited snapshot”?

>> Thanks, corrected now.

Referee #2 (Remarks on code availability):

The published code is completely inadequate, with no documentation whatsoever, and I cannot even find where POPout is implemented.

>> We apologise for the state of our code on initial submission. We have now produced a user-friendly software written in Python and R to allow users to run our POPout test on their own data. We have built a new dedicated website (www.tailstests.org) to host our software, which provides download links and clearly describes required packages, user options, input file formatting and output files. The website includes a quickstart guide and simulated toy data from five different traits so that users can begin running POPout immediately.

Referee #3 (Remarks to the Author):

This manuscript is interesting in that it focuses on the behaviors at the interesting tails of trait distribution, which is arguably most relevant for diseases in contrast to the normal healthy range of traits. The authors point out the interesting distribution range of traits that is closer to Mendelian diseases rather than complex diseases. Although interesting, the concepts are not brand new and the importance of identifying and studying rarer alleles with larger effects has already been appreciated since about two decades ago. The progress has been largely limited by sample ascertainment and sequencing resources for certain population groups. The authors have carried out careful analyses but some of the analyses are simplistic and somewhat repetitive.

>> We absolutely agree that the importance of identifying and studying rare alleles with larger effects has been long appreciated and we regret that the framing of our paper indicated that we may be unaware of this. We have now completely overhauled the framing of our paper to make this appreciation clear and we also now clarify that the key advances offered by our paper are: (1) an evaluation of the *extent of impact* of rare variants in the tails of a large number of complex traits (note that even for the same trait, we observe evidence for a potentially substantial impact in one tail but not the other, which was not previously known for these traits), (2) provision of novel population and family-based tests to *infer* tail architecture from genotype-only data (population) or trait-only data (family). Even when a study sample includes WGS data, identification of rare variants is severely limited by statistical power, which means that such studies have typically provided weak insight into tail architecture, (3) convincing evidence, both from real and simulated data analyses, that selection is the mechanism driving the widespread departures from common polygenic expectations observed in trait tails.

I also have quite a few specific questions and comments.

Page 4, lines 111-112, QC step for "the middle 80 trait centiles": how many traits were removed based on this criterion?

>> None of the UKB traits in the primary analysis were removed due to this criterion. This was due to a combination of the large sample size of the primary UKB analyses reducing stochastic variation and because one of our preliminary QC filters was for oligogenicity (defined here as being where one SNP explains > 2% trait variance). Had this filter not been present, then three traits (30660 [Direct Bilirubin], 30840 [Total Bilirubin] and 30790 [Lipoprotein A]) would have failed this QC. However, in our smaller sample sized replication analyses multiple traits were removed based on this criterion: 13 of 63 overlapping traits with repeat-measures, 9 of 63 traits in the multi-ancestry replication, and 5 of 33 overlapping traits in All of Us. We have now added these details to the Methods.

Page 4, line 114: I am also curious to see how results would vary with different definitions/thresholds of extreme values, especially given some complex diseases have much higher prevalence than 1%.

>> We absolutely agree with the reviewer that it is important to get a better idea of results at different tail thresholds and, as such, we have addressed this point in three different ways: (1) we have repeated the primary analyses in UKB data across all 74 traits at tail thresholds of 10%, 5%, 0.5% and 0.1%. The results, which are presented in Supplementary Figure 2 and discussed in the main text, and include comparison with the 1% threshold results, show largest mean POPout effects at the 0.1% tail threshold and the greatest number of significant results at the 1% threshold. Results at the 10% threshold, which may be reflective of more prevalent diseases, show the smallest POPout effects and the fewest significant results, which may reflect the consequences on the population if a large fraction of the population were subject to severe selective constraint; (2) we have interrogated our rare+common PRS results at ten thresholds: 1%, 0.9%, 0.8%, …, 0.1%, to fully investigate the behaviour of results within the 1% tails, where much of the POPout effects occur (see above). These results are presented in Supplementary Figure 4 and discussed in the main text; (3) we have updated our POPout analysis software (www.tailstests.org) to allow users to specify the tail threshold at which to perform POPout testing.

Page 6, line 134: The "large, non-Gaussian, unadjusted environmental risk factors" could also offer a potential explanation of why most trait tails demonstrate departure from polygenicity beyond the offered hypothesis of rare allele enrichment. It's possible that large unadjusted environmental factors drive the trait values toward the tails and they cannot be captured by PRS, resulting in the observations that tail trait values may show PRS towards the mean. This potential explanation undermines the value of the central hypothesis proposed here: primarily due to rarer variants with larger effect sizes.

>> While we agree that the environment has likely played an important role in these observations, we do not think that this conflicts with our central hypothesis – that high-effect, rare alleles are enriched in complex trait tails and that this is largely driven by the effects of selection. We think that we have provided compelling evidence supporting this hypothesis across a range of real data approaches and through rigorous simulation. To limit the effects of the environment in our analyses, we controlled for multiple environmental risk factors directly, performed replication across repeat measures and between

cohorts from different continents with different environments (UK Biobank, All of US), applied different approaches (population / family based) and performed selection analyses – using both simulated and real data – suggesting that selection leads to an enrichment of high-effect, rare alleles in trait tails. In our new WGS analyses, we also now provide direct evidence for the contribution of rare variants to POPout effects. Together, we believe, that these results provide strong evidence that rare variant enrichment is a key explanation for our observations, and we also provide a comprehensive evaluation of the *extent* to which rare variant tail enrichment is present across a large range of traits (including showing that some traits appear to have no/little such enrichment).

Nevertheless, we agree with the reviewer that environmental causes of our observations are important to acknowledge and consider. To address this, in our new analyses that estimate the heritability that could be explained by unidentified rare variants (Fig.4d) we also provide estimates based on the environment explaining a fraction of POPout effects that corresponds to (1 – heritability). Moreover, we also now further acknowledge and highlight the importance of environmental contributions to our observations in the Discussion.

Page 6, line 139: “disease prevalence of 1%” It's by nature more difficult for PRS to predict rarer yet polygenic diseases. Is this pattern also observed for traits with tails not demonstrating departure from polygenicity, e.g., phosphate, as a negative control mentioned by the authors? I am also wondering if under-powered PRS performance for some complex diseases could be explained by this tail departure. As the authors showed in the dichotomized tail behaviors, top 1% showed poor performance while top 25% showed expected performance. Along with this, it would be interesting to more systematically evaluate the relationship between PRS performance for complex diseases and their estimated prevalence to see if there is any relationship.

>> To address this point, we have now expanded our analyses of PRS accuracy to the other index traits - phosphate, as a negative control, and also sitting height, in which POPout is identified in both tails. We do find that the same pattern is also observed for these traits – i.e. the PRS for phosphate shows strong prediction when analysed as a hypothetical disease with prevalence 1%, as expected, and likewise for a prevalence of 25% (higher ORs in the 1% case for those with highest PRS). Sitting height, which shows POPout effects in both tails, shows reduced PRS prediction in both tails compared to expectations, especially when considered as a disease of prevalence 1%. We also note that Red Blood Cell Distribution Width and Haemoglobin each act as their own internal controls, since for each we expect reduced PRS performance in one tail (with POPout effects) but normal performance in the other tail, which is what we observe. We agree with the reviewer that it would be extremely interesting to know if our findings explain reduced performance of PRS in complex diseases, but given the multi-factorial nature of PRS performance (including GWAS power, disease heritability, disease misspecification, sample vs population prevalence), as well as our POPout method not being presently suitable for application to binary outcomes, we plan to investigate this in our future work, which will focus on the impact of our findings on disease more specifically.

Our expanded analyses of the impact of POPout effects on PRS accuracy are presented in Supplementary Figure 1 and discussed in the main text.

Page 6, line 156: It seems the authors performed UKB Multi-Ancestry analysis combing all the ancestries together. Do the authors consider ancestry as a potential confounder of tail behaviors, especially given some traits have different mean, variance and extreme values across ancestries, and rich literatures have shown PRS had varied performance across ancestries? I would suggest performing replications separately for each population, perhaps in AoU, which has larger non-EUR sample sizes than UKB.

>> This is correct, we performed UKB Multi-Ancestry analysis by combining all ancestries together, and we now make this point clearer in the main text to ensure that readers understand that this is what was done. We do not believe that ancestry is a confounder since we do not think that heterogeneity in ancestry could have produced, and replicated, the POPout effects that we observed in the primary analysis in European ancestry individuals; differences in PRS distributions may have meant that some of the ancestries contributed more to the mean POPout effect than others, but this combination of different PRSs cannot have produced false-positive POPout signal. Unfortunately, the sample sizes of different ancestry groups in the UK Biobank does not permit discovery POPout analyses in the separate ancestries (see Supplementary Figure 3c) that would allow a meaningful replication of those stratified ancestry analyses in the All of Us data. However, our multi-ancestry replication analyses in the UKB provide strong support for POPout effects being present in these traits across different worldwide ancestries (as well as different geographical regions given our All of Us replication), and we hope that our study motivates discovery POPout analyses in the emerging large-scale cohorts and biobanks comprising different ancestries throughout the world.

We thank the reviewer for this point and have now updated our main text, Methods and Discussion to clarify how our multi-ancestry replication was performed, our consideration of confounding in this context, and to expand on our study limitations and future prospects in relation to evaluating POPout effects across different ancestries and populations.

Line 199: The authors hypothesized that one explanation of trait tails demonstrating departure from polygenicity is enrichment of rare alleles in the tails. They further used WES associations from a previous study to validate their hypothesis, where the results show significant but modest correlation between the amount of tail departure and the number of WES signals. Given that UKB has WGS data available now, it would be much more compelling to validate with WGS associations. In addition, the authors only looked at associations between number of WES signals and amounts of tail departure. It would be more interesting to see if such departure could be mitigated after accounting for these rare alleles to further confirm.

>> Following this point we have now performed rare variant analyses of the WGS UK Biobank data, enhanced by the fact that the 500k data became available in a workable format (plink) during the period in which we were preparing responses to these reviews. We first performed variant-by-variant association testing among rare variants down to a MAF of $1x10^{-4}$ across all 74 QC'ed traits and in the same half of the UKB sample as previously used for common-variant GWAS. Next, we aggregated the significant variants from these analyses to compute a rare-variant PRS for each trait in the other (test) half of the UKB and added this to our existing common-variant PRS. We then evaluated any reduction in the POPout signal from inclusion of these 'intermediate' rare variants. This approach constitutes a highly tractable, replicable and robust way to incorporate rare variants into our PRS and test for any reduction in POPout effects. Our results show that among 68 trait tails with significant POPout and a corresponding rare PRS for testing (i.e. with at least one significant rare variant), 49 had reduced POPout ($P = 6.5x10^{-5}$), 7 had significantly reduced POPout with a mean reduction in POPout effect of 27% and 0

had significantly increased POPout (Fig.4c, Fig.4d, Supp.Fig.4). While the inclusion of these rare variants in the PRS increased trait variance explained by the PRS only modestly, the increase in odds ratio relating to extreme trait status is substantial (Supplementary Table 2), confirming the importance of rare variants in prediction of extreme trait values. Moreover, a large fraction of the rare variants that we identified and included in the rare PRSs were present in the ClinVar database (67 of 609 unique variants; see Supplementary Table 4), providing reassurance that our WGS analyses were robust and identified variants with established effects on the relevant traits. Thus, overall we find direct evidence for the contribution of rare variants to the observed departures from common polygenicity in the tails of our tested traits and demonstrate mitigation of the effects after accounting for these rare alleles.

Line 245: "proxy measures of fitness": It would be helpful to provide more intuitions of why such associations are expected and why these outcomes are selected as proxy measures of fitness.

>> We have now described our intuition about the selection of these measures and their expected associations in the main text and, on reflection, we feel that describing them as "proxy" measures of fitness was too strong and so we have now described them as simply being related to fitness.

Line 354: It seems the authors only used ~800K genotyped markers without considering imputed data. I am wondering if the tail departure could be explained or mitigated by untyped common variants.

>> PRS based on additional variants imputed from the ~800K genotyped markers would be highly correlated with our original PRSs; Nguyen et al. 2022 [PMID: 36266455) estimate the correlation between PRS based on imputed data and the UK Biobank Axiom 844k genotyping array to be > 0.95 for all major ancestries (>0.98 for the European ancestry of our primary analyses). Thus, imputed SNPs would not be able to explain the highly significant and substantial deviations from common polygenic expectations that we observed across the traits (noting that uncertainty in our expectations is accounted for).

Line 361: The authors applied MAF 1% to calculate PRS for common variants. While 1% is a commonly used cutoff for common variants, given the large sample size of UKB, many PRS analyses in literature went beyond the thresholds to capture more signals. I am wondering how the results would change if relaxing the threshold, e.g., to 0.1% to include some low frequency variants in PRS. It may strengthen the claim that rare allele enrichment leads to tail departure from polygenicity if such trend was mitigated.

>> Following the reviewer's point here and above, we have now performed a WGS analyses of the UKB data, analysing and incorporating rare variants down to a minor allele frequency of 0.01% (i.e 10x lower than the suggested 0.1%), which demonstrated mitigation of effects as described in detail above. This is a major addition to our manuscript, substantially strengthening support for our central hypothesis and improving the overall scientific quality and rigor of our work, and so we are extremely grateful for the reviewer for highlighting the benefit of performing such analyses to our manuscript.

Line 379: a distribution would be helpful to make sure there are not a lot of pairs at IBS0 above but close to the threshold.

>> To select siblings, we used the same method as used in Cheeseman et al 2020. in application to UKB data. See Figure 1 in Cheeseman et al. 2020, which shows the Kinship plotted against IBS0 for all first-degree relatives in the UKB, confirming clear delineation above and below the applied threshold. We have now clarified this point in the Methods section.

Line 579: Only using BIC to select models may correspond to two scenarios: (1) the selected model is much more favored over others; (2) the selected model performs very similar to another model but is randomly selected. Thus, I would suggest some sensitivity analyses: for each trait, recording the two models with highest BICs, perform a likelihood ratio test to see if the final model (higher BIC between the two) is significantly better than the other one.

>> We have now performed a sensitivity analysis in relation to our BIC models as suggested by the reviewer. We first distinguish our models into the two high and low confidence models that the reviewer refers to. 57 of our 74 traits are high confidence models, in which the selected BIC model fits the data significantly better than all the other models. 17 of the 74 models are low confidence models, in which the selected BIC model is not significantly different from the second-best model. Next, we repeated our entire analysis comparing POPout effects with selection types, this time replacing the low confidence models with the second best-performing model, to test the sensitivity of our results to the BIC selection procedure. Our repeated analysis – presented in Supplementary Table 6 – shows extremely similar results to our original results. As previously, there were no significant tail differences in POPout effects among neutral and non-directional stabilising selection models, significantly larger lower tail POPout effects for traits with positive selection models (P = 2.1x10^{-5} vs P = 7.8x10^{-7}) and significantly larger upper tail POPout effects for traits with negative selection (P = 0.03 vs P = 0.02). These new analyses are highlighted in the main text, described in detail in the Methods and the corresponding results are presented in Supplementary Table 6.

We thank the reviewer for this point as we believe that this sensitivity analysis has substantially improved the robustness of our analyses linking our observations with the effects of selection.

Referees' comments:

Referee #1 (Remarks to the Author):

The authors did an excellent job with revising their manuscript. In particular, they have added a number of new and substantial analyses which buttress their original claims. They have also performed some robustness checks on a number of key analyses. Lastly, the framing of the paper is much improved to be better in line with the existing literature.

I believe this is an important study, worthy of publication.

That said, I have a number of suggestions on things to improve. Many of these comments stem from the new analyses that the authors included in the revised manuscript.

1. Title: Overall, the authors did a nice job with framing the manuscript better in terms of the existing literature. The current title now reads, “Striking departures from common polygenicity in the tails of complex traits”. I’m afraid that this phrasing seems awkward and ambiguous to me. My particular unease stems from the “Common polygenicity” phrase. I would suggest the authors try to find different words to say what they really mean, rather than leaning on the jargony “polygenicity”. Some alterative titles could be, “Striking departures from common variant architectures in the tails of complex traits” or “Large-effect rare alleles drive the tails of complex traits”.

>> We agree that the term “polygenicity” is best avoided, since it is jargony and relatively ambiguous. We agree that “common variant architecture” would be much better, and so we have now changed the title and text accordingly throughout.

2. Line 8 (and elsewhere): Similar to my comment above, I suggest using something other than “common polygenicity” to describe this phenomenon of rare alleles of large effect influencing phenotypes in the tails.

>> See above.

3. Line 41: “…contrasting expectations under neutrality…”. “Expectations” by itself is a little awkward. Expectations of what? Consider rephrasing.

>> We have now changed this phrase to “based on our different expectations of genetic architecture under neutrality and selection”.

4. Lines 246-249 and Figure 4d: I had a really difficult time following most aspects of the rare variant heritability analysis. Lines 246-248 outlines the results for the heritability calculated using only common variants and for the heritability accounting for non-genetic factors. What about when including rare variants? That result isn’t mentioned din the text here. I thought this would be the main result, so its absence in the Results is a little odd.

>> Apologies for the lack of clarity here - in Figure 4d the results of including rare variants are shown in green (“Including WGS variants”), while in the text we should have made clear that the estimate of heritability accounting for non-genetic factors also accounted for rare variants (we considered this our best estimate of remaining rare-variant heritability, but should have stated this). However, we have now removed the rare variant heritability analysis from the manuscript. This is because (as explained to reviewer 2 in response to their feedback on this analyses): (1)

it detracts from the main focus of the paper on deviations in the tails, rather than estimating h2, (2) there are other significant publications devoted specifically to estimating h2 (e.g. Weiner et al 2023, Wainschstein et al 2025), (3) further development of our modeling will be required to partition the effects of de novo mutations, rare variants and environmental factors in contributing to POPout effects, which we think is best performed as part of devoted work in a follow-up study.

5. Figure 4d: Related to my comment above, if I understand things correctly, it appears that the heritability estimates are slightly lower when including the rare variants compared to when only using common variants. This would seem to go opposite what I would expect to be happening under the authors' hypothesis. Thus, I am concerned that this heritability analysis includes some unrealistic assumptions or is biased in some other way.

>> The heritability estimates reported were remaining heritability still to explain, which is why these were lower after inclusion of rare variants. However, as explained above, these analyses have now been removed from the manuscript.

6. Figure 5e and associated discussion: Testing for an association of the POPout scores and particular traits related for fitness is clever. However, the description of this analysis provided in the legend for Figure 5e, the main results, and even the Methods section is lacking in clarity and details. Some of the confusion, I think, stems from stating (e.g. lines 808-809) that the "mean value was calculated separately in the upper and lower tail for every trait." It's not clear what "trait" refers to here. If I'm understanding this, the authors took the upper tail of one of the 5 fitness traits, and then computed the mean value for each of the 74 other UKBB traits that they computed POPout on. They then tested whether the POPout static for the 74 UKBB traits was correlated with differences in that trait co-varying with differences in fitness. Another question is how the "lower and upper tail means" were tested for the correlation with POPout. How was the difference in means between the tails tested? Or the lower and upper tails separately? Why? More description is needed. Maybe a diagram could help as well.

>> Apologies for the unclear descriptions of this test. We have now re-written the related sections of the Figure 5 caption, main text and Methods to clarify how these tests were performed. Briefly, and as now stated in the main text, "Spearman's rank correlations were computed, for each fitness measure, between the mean fitness value of individuals in each tail of each trait (e.g. mean paternal age in both tails over the 74 traits) and the corresponding POPout effect."

7. There are a number of related references that would be good to include regarding selection acting on complex traits decreasing the portability of PRS. These include: PMID: 40499537, PMID: 33691092, PMID: 35430887, PMID: 35522704.

>> We have now added references PMID 33691092 and PMID 35430887 to our discussion of PRS portability (in the Discussion), since these best-support our statements on PRS portability and were omissions given that we are highly familiar with these papers given their important connections with our work here. We have also added reference PMID: 40499537 in relation to a point in the Discussion relating to selection and mutational target sizes, given its relevance to this in relation to the loss of alleles of large effect due to historical stabilising selection.

Referee #2 (Remarks to the Author):

In their revised manuscript, Souaiaia et al. reframe their findings around the following hypothesis:

>Based on the existence of rare alleles that can alone generate extreme trait values, and growing evidence that stabilising selection is a pervasive driver of complex trait genetic architecture, we hypothesise that stabilising selection on some traits may be strong enough to have produced marked enrichments of high-effect, rare alleles in the tails of continuous, complex traits.

This addresses my previous concern that tail enrichment is a known phenomenon which does not contradict existing models. Also, the authors have thoroughly addressed my comment about code availability.

I still have major comments about the revised manuscript. Comments 1-2 are related to the new framing, and comment 3 is related to the new heritability analysis.

Major comments

1. A subtle point bothered me when reading the revised manuscript. We know that trait-associated variants are under negative selection, and we know this because large effect alleles are rare. The rarity of large effect alleles is what causes tail enrichments. When the authors state that tail enrichments are caused by negative selection, this is true, but there is no causal arrow from negative selection to tail enrichment that does not go through allele frequencies – and allele frequencies are themselves the evidence we have for selection. All believable models of selection on traits already predict that large-effect alleles are rare and would therefore also predict tail enrichment. On the other hand, it is still of interest to identify novel consequences of selection even if they do not help us to distinguish between competing models. I suggest that when introducing the hypothesis that selection explains tail enrichments, the authors explicitly lay out that selection directly affects allele frequencies, and it is the rarity of large-effect alleles that causes tail enrichments.

>> We have now more explicitly clarified, in the first two paragraphs of the Introduction and also the first paragraph of the Discussion, that it is the impact of selection on the allele-frequency/effect-size relationship that leads to tail enrichments. These clarifications complement Figure 1, which illustrates this point. We also make clear (2nd paragraph of Intro) that the extent of tail enrichments is governed by the effect sizes, rarity and also the number of these high-effect alleles.

We agree that the key advances here relate to novel *consequences* of selection, although we do think that this work could contribute to future modelling of selection by enhancing inference of the effect-size/frequency relationship, particularly at the ultra-rare end that involves effects that are difficult to identify or infer the sizes of directly. We refer to this point in the Discussion (2nd paragraph).

2. Relatedly, now that selection is a central theme of the paper, the authors should be careful about different models of selection. In the Introduction, the authors frame their hypothesis around a particular model, direct stabilizing selection, and state that this model fits their data better than a neutral model (this is convincing). Later on, they show that alternative models involving directional selection fit their data better for particular traits. I do not really recommend repeating the simulations under these various models. Instead, I recommend editing the text in

two ways: (1) to clarify that there exist various modes of selection that could produce similar results; and (2) to distinguish between selection on trait-associated alleles vs. selection on traits (i.e., pleiotropic vs direct selection). I don't think the current handling of pleiotropy in the Discussion really helps with this point.

>> We should have previously clarified that our illustrative example relating to stabilising selection discussed in the Introduction and depicted in Figure 1, can be reasonably well generalised to other realistic forms of selection on complex traits (investigated later in the paper). We also agree that discussion of selection acting on pleiotropic variants is needed early in the manuscript. We have now clarified our perspective on both of these issues in the Introduction (end of 2nd paragraph).

3. The new calculation of h2_her is interesting, but the estimates are implausible.
They are definitely too large – I think if you compared them with common variant estimates, they would imply rare h2 >> common h2 for many traits, and probably they would imply common h2 + rare h2 >> twin h2. Worse, it looks to me like the largest estimates correspond to the least heritable/least powered traits, such as glucose levels (non-fasting glucose is famously noisy) and age at menopause (which has 50% smaller sample size). Height, definitely the best-powered and most-heritable, has a much smaller estimate; but for some reason, sitting height has a much larger estimate.

The procedure that estimates h2_her is intricate, and I can see any number of reasons that it could fail. My guess would be that statistical noise in the POPout statistics causes bias in the heritability estimate. I recommend either dropping this estimator from the manuscript or performing extensive validation + troubleshooting analyses.

>> We should have made the assumptions of our h2_her estimates clearer. In particular, we made the simplifying assumption that the unobserved trait distribution, removing rare large effect variants, y' follows a Gaussian distribution with mean zero and variance one. While this is a good approximation when the POPout effect is relatively small, when there are larger POPout effects the estimates become inflated. While we have since developed a simulation procedure that better models the distribution of y', we think that it would be best to drop this estimator from the manuscript as proposed by the reviewer because: (1) it detracts from the main focus of the paper on deviations in the tails, rather than estimating h2, (2) there are other significant publications devoted specifically to estimating h2 (e.g. Weiner et al 2023, Wainschstein et al 2025), (3) further development of our modeling will be required to partition the effects of de novo mutations, rare variants and environmental factors in contributing to POPout effects, which we think is best performed as part of devoted work in a follow-up study.

This analysis was intended to address my previous question about whether POPout effects are explained by known rare variant effects, in particular the burden heritability. I liked the analysis where the authors conditioned on a rare-variant PRS. I recommend expanding this analysis to include exome-wide significant burden statistics; I note that for many traits, such as height and LDL, roughly half of burden heritability is explained by exome-wide significant genes that can be included in a PGS.

>> We have now expanded our rare variant analyses to the UK Biobank Whole Exome Sequence data to identify significant 'burden variants', and compute corresponding burden variant PRSs and enhanced rare+common PRSs. This has resulted in substantial further reductions in observed POPout effects (as shown in our revised Figure 4), and corresponding marked increases in prediction of phenotypic extremes (summarised by odds ratios relating to

tail status). This is despite: (1) our exclusion of any variants in the MAF range 0.01% - 1% that we had already identified and included in the ‘rare PRS’ previously, and (2) power being limited here by performing discovery on half the UK Biobank (with POPout calculated in the other half), (3) not incorporating non-coding, ultra-rare variants.

Minor comments

4. In derivation of the h2^her estimator, the authors skip a key step in the derivation between equation (2) and the following equation – it looks like there are two terms, one involving y and the other involving y’, which are assumed to cancel out. I believe that this is actually correct but non-obvious, and it should be spelled out and justified if this estimator is retained in the text.

>> Estimator no long retained, as discussed above.

5. I should have flagged this before; “positive selection” and “negative selection” on traits should be called “positive directional selection” and “negative directional selection” (“positive selection” being a type of selection that acts on an allele).

>> We have now revised this terminology accordingly throughout the text.

6. The new Discussion sentences about the mutational target size somewhat miss the point of my previous comment. Instead of being a limitation that target size is not considered, I’d call it a missed opportunity: suppose we already knew that large-effect alleles can only be rare, but we didn’t know if they can exist at all. Maybe there is no mutation in the genome that causes a 10cm effect on height. POPout would tell us that such mutations can indeed occur; it tells us something potentially new about the mutational target.

>> We agree that this would be extremely interesting to gain insight on as well and we considered this as part of the h2 estimator (above) that we developed. It is not straightforward to estimate mutational target sizes from POPout effects because there is at least some degree of non-identifiability, in that a given POPout effect size can be caused by rare variants corresponding to different combinations of effect sizes, allele frequencies and number across the genome. However, we have now highlighted the possibility of making inference about mutational target sizes from POPout effects with future model development (2nd paragraph of Discussion), and so thank the reviewer for this point. We have now removed the discussion relating to target size as a study limitation.

7. Regarding training the PGS and running POPout in the same individuals, is it true that this is done in UK Biobank but not in ‘UKB multi-ancestry’ and AoU?

>> Yes, this is the case and this should have been previously stated. We have now detailed how these out-of-sample replication POPout analyses were performed in the Methods section “Replication of POPout associations”.
